# Trophoblast-derived Lactic Acid Orchestrates Decidual Macrophage Differentiation via SRC/LDHA Signaling in Early Pregnancy

**DOI:** 10.7150/ijbs.67816

**Published:** 2022-01-01

**Authors:** Lu Gao, Qian-Han Xu, Li-Na Ma, Jing Luo, Kahindo P. Muyayalo, Li-Ling Wang, Dong-Hui Huang, Xian-Jin Xiao, Shi-Bin Cheng, Gil Mor, Ai-Hua Liao

**Affiliations:** 1 Institute of Reproductive Health, Center for Reproductive Medicine, Tongji Medical College, Huazhong University of Science and Technology, Wuhan 430000, P.R. China.; 2 Departments of Pediatrics, Obstetrics and Gynecology and Pathology, Women and Infants Hospital of Rhode Island, Warren Alpert Medical School of Brown University, Providence, RI 02905, USA.; 3 C.S. Mott Center for Human Growth and Development, Wayne State University school of Medicine, Detroit, MI 48201, USA.

**Keywords:** lactic acid, trophoblast, early pregnancy, decidual macrophage, recurrent pregnancy loss

## Abstract

Lactic acid (LA) metabolism in the tumor microenvironment contributes to the establishment and maintenance of immune tolerance. This pathway is characterized in tumor associated macrophages. However, the role and pathway of LA metabolism at maternal-fetal interface during early pregnancy, especially in decidual macrophage differentiation, are still unclear. Herein, for the first time, we discovered that LA can trigger either M2 or M1 macrophage polarization via oxidative phosphorylation and glycolysis regulation under normoxia or hypoxia, respectively. Also, LA metabolism played a vital role in decidual macrophages-mediated recurrent pregnancy loss (RPL), through HIF-1α/SRC/LDHA pathway. Moreover, blockade of LA intake with AZD3965 (MCT-1 inhibitor) could rescue pregnancy in an abortion-prone mouse model, suggesting a potential therapeutic target in RPL. Collectively, the present study identifies the previously unknown functions of LA metabolism in the differentiation of decidual macrophages in early normal pregnancy and RPL, and provides a potential therapeutic strategy in RPL by manipulating decidual macrophages' functions through LA metabolic pathway.

## Introduction

During normal pregnancy, macrophages are important participants in maternal-fetal immune tolerance. Also, decidual macrophages are critical in embryo implantation, spiral artery remodeling, and placentation 1. Macrophages are roughly categorized into two different subsets, namely, classically activated (M1) and alternatively activated (M2) macrophages 1, 2. The M2/M1 ratio in the decidua increases in early normal pregnancy, and a decrease of this ratio is associate with adverse pregnancy outcomes, including recurrent pregnancy loss (RPL) and spontaneous preterm birth 1, 3. In line with other studies, our previous results indicated that decidual macrophages showed distinct differences from conventional macrophages and exhibited dynamic polarization changes during pregnancy 1-4. Several factors regulating M1 versus M2 polarization during pregnancy have been found, including growth factors, cytokines, chemokines, hormones, Notch signaling, infection, as well as negative co-stimulatory molecules 1, 2, 4. Recent studies have shed light on the interconnection between metabolism and immunity in multicellular organisms and their functional coordination for the effective establishment and resolution of immune responses 5, 6. However, whether the metabolic changes occurring in the human decidua may also influence macrophage polarization in early pregnancy is still unclear.

Lactic acid (LA) is a metabolic product produced from glucose through glycolysis and the conversion of pyruvate by lactate dehydrogenase (LDH) under oxygen deprivation conditions 7, 8. Studies have proven that tumor-derived LA regulates macrophage polarization through the G protein-coupled receptors (GPRs) and the monocarboxylic acid transporters (MCTs) -mediated “lactate shuttle” 9. Indeed, acidification of the local microenvironment by tumor-derived LA promoted the conversion of tumor-associated macrophages to the M2 phenotype 10. Conversely, a reduction in LA could inhibit tumor growth and M2 macrophage polarization 11. In early pregnancy, a high LA microenvironment is required for human embryo development *in vivo* 12, 13. LA can regulate physiological processes, such as promoting the decomposition of endometrial tissue, inducing angiogenesis, increasing vascular permeability and promoting immune tolerance 7, 8. Prior studies have already shed light on enhanced LA metabolism in early pregnancy in mice 12, 13. However, the effect of LA metabolism on macrophage polarization at the maternal-fetal interface has not yet been elucidated.

During early pregnancy, the uterus requires a transition of oxygen saturation for normal embryonic implantation and further development in the second trimester 14. This means that the embryo needs to adapt to the metabolic microenvironment change during early pregnancy. The embryo exhibits idiosyncratic metabolism characterized by utilization of glucose as the main nutrient through both oxidative and aerobic glycolysis, a metabolic characteristic of tumors 15, 16. This metabolic feature reflects the embryo's unique physiology and its ability to undergo implantation. In the pre-implantation state, the inner cell mass (ICM) gives rise to the embryo accompanied by the interconversion between oxidative phosphorylation (OXPHOS) and glycolysis 17. In hypoxia, this metabolic switch from OXPHOS to glycolysis is induced by regulators of oxygen homeostasis called hypoxia-inducible factor (HIF) family (mainly HIF-1α and HIF-2α) 18. LA is an essential metabolite in HIF-mediated glycolysis 18. Under normoxia, LA is oxidized into pyruvate by lactate dehydrogenase B (LDHB) and thereby supports pyruvate-mediated HIFs activation. In hypoxia, LA, synthesized by LDHA, which is transcribed by highly expressed HIF-1α, can work as an active metabolite via a MCT-mediated shuttle (mainly MCT-1 and MCT-4) in immune and cellular biological regulation under physiological and pathological conditions. Recent studies have highlighted the cellular metabolism shapes the immune cell activation and differentiation into distinct cellular states, especially macrophages 5. However, whether trophoblasts artificially secret LA and its effects on the macrophage polarization under normoxia and hypoxia remains largely unclear.

In the current study, we focused on elucidating the regulatory effect and underlying mechanism of LA metabolism in decidua on macrophage polarization in women with normal pregnancy (NP) and RPL. We observed that decidual macrophages in women with NP and RPL were polarized to M2 and M1 phenotypes, respectively. Moreover, LA production through anaerobic glycolysis was enhanced in the decidua of women with RPL, and the trophoblasts might be the main LA source both in women with NP and RPL. Importantly, trophoblast-derived and exogenous LA could regulate pregnancy-associated macrophage polarization to either M2 or M1 phenotypes under normoxia or hypoxia, respectively. The underlying mechanism is related to the metabolic reprogramming in HIF-1α independent under normoxia or HIF-1α dependent manner under hypoxia via the SRC/LDHA pathway, which leads to the increased expression of vascular endothelial growth factor (VEGF) and inducible nitric oxide synthase (iNOS), respectively. More importantly, blocking LA intake with MCT-1 inhibitor (AZD3965) could rescue the pregnancy in abortion-prone (AP) mice. Therefore, our findings reveal for the first time that enhanced trophoblast-derived LA in the decidua of women with RPL may trigger M1 macrophage polarization driven by the HIF-1α/SRC/LDHA pathway.

## Materials and Methods

### Human sample collection and ethical statement

Human first-trimester villous tissues and peripheral blood were obtained from healthy pregnant women (age: 32.33 ± 3.35 years; gestational age: 8.15 ± 0.56 weeks) for primary trophoblast and macrophage studies, respectively. In addition, human first-trimester decidua from healthy pregnant women (n = 24) and RPL (n = 24) (age: 32.02 ± 2.30 years, diagnosed as more than two spontaneous abortions at 7.77 ± 0.43 gestational weeks) were collected for immunofluorescence and western blotting analysis. Normal pregnancy was electively terminated for nonmedical reasons. Women with RPL were terminated the pregnancy by the medical issues. All subjects gave informed written consent for the collection and study of tissue samples. This study protocol was approved by the Human Research Ethics Committee of Tongji Medical College, Huazhong University of Science and Technology, Wuhan, China.

### Animals

Experimental protocols were approved by the Animal Ethics Committee in Huazhong University of Science & Technology. BALB/c males, DBA/2 males and CBA/J females mice (6-8 weeks) were purchased from Shanghai Jihui Laboratory Animal Care Co.,Ltd (Shanghai, China, animal license: SCXH2017-0012). All mice were bred at the Experimental Animal Center of Tongji Medical College, Huazhong University of Science and Technology (Wuhan, China) in pathogen-free conditions. Male BALB/c mice were mated with female CBA/J mice as a normal pregnancy (NP) group. Besides, male DBA/2J mice were mated with female CBA/J mice as an abortion-prone (AP) group. The morning of detection of the vaginal plug was considered as E0.5. The mice were divided into 5 subgroups, namely the NP control group (treated with PBS), AP control group (treated with PBS), and AP intervention groups treated with low (L, 25 mg/kg, *bid*), moderate (M, 50 mg/kg, *bid*) and high (H, 100 mg/kg, *bid*) dosages of AZD3965 (MCT-1 inhibitor, A14186, Adooq Bioscience, China), respectively. All the mice were orally administrated at E5.5 and euthanized at E10.5. The resorption embryos were identified by their smaller size and darker appearance. The embryo resorption rate was calculated (number of resorptions/total number of formed fetuses and resorptions) × 100%.

### LA measurement

Human decidual tissues were washed and homogenized with cold PBS buffer containing 1 mM PMSF. Then, the supernatant was collected after centrifugation at 12,000 × g speed for 10 minutes at 4 °C. The BCA method was used to determine the protein content, and the LA content was evaluated using the manufacturer's protocol (Abcam, ab65331) with the following changes. Before the measurement, 100 μL of sample was heated at 95 °C for 5 minutes prior to the assay to inactivate any endogenous enzymes. Data are presented as sample LA concentration (mM/g) normalized to tissue wet weight. Primary trophoblasts were seeded in 6-well plates at a density of 3 × 10^5^ cells or 1 × 10^6^ cells/well and incubated in an atmosphere of 21% O_2_ and 1% O_2_ for 24 h, 48 h, 72 h and 96 h. The cell culture supernatants were collected and used to measure the levels of secreted LA according to the manufacturer's instructions.

### Immunofluorescence

The human decidua was fixed in formalin immediately, followed by standard paraffin embedding. Immunofluorescence staining was performed overnight with the designated primary antibodies: anti-CD68 antibody (Abcam, ab31630), anti-CD206 antibody (Abcam, ab125028), anti-CK7 antibody (Servicebio, GB12225), anti-LDHA antibody (Abclonal, A1106), anti-CD86 antibody (Abclonal, A1199), anti-MCT-4 antibody (ProteinTech, 22787-I-AP) for 12 h at 4 °C. Secondary fluorescent antibodies: CoraLite594-conjugated goat anti-mouse IgG (ProteinTech, SA00013-3), fluorescein (FITC)-conjugated Affinipure goat anti-rabbit IgG (ProteinTech, SA00003-2), were added for 2 h, and DAPI (Servicebio, G1012, 1: 5000) was used for nuclear counterstaining. Samples were imaged through an immunofluorescence microscope (Olympus, Japan) 24 h after mounting.

### Primary trophoblast culture

Fresh first-trimester villi were immediately collected under sterile conditions in ice-cold phosphate-buffered saline (PBS) supplemented with antibiotics (100 IU/mL penicillin and 100 g/mL streptomycin). Collected specimens were immediately transported to the laboratory for cell isolation as previously described 19. Briefly, the trophoblasts were isolated by 0.25% trypsin digestion at 37 °C with gentle agitation for 10 min/cycle over four cycles and discontinuous Percoll gradient centrifugation. Then the cells were cultured in DMEM-high glucose complete medium (2 mM glutamine, 25 mM HEPES, 100 IU/mL penicillin, and 100 μg/mL streptomycin) supplemented with 10% heat-inactivated FBS and incubated in a 5% CO_2_ atmosphere at 37 °C. The trophoblasts were identified with cytokeratin7^+^ (CK7^+^) staining by flow cytometry and immunocytochemical staining. CK7^+^ cells at passages 3-5 with the purity over 90% by flow cytometry were used in the experiment. Trophoblast-conditioned medium (TCM) was obtained from the culture supernatant of the trophoblasts cultured for 48 h at a density of 1×10^6^ cells/ml. Cell supernatant was collected, aliquoted, and stored at -80 °C until use.

### Macrophage culture

Macrophages were isolated and cultured as previously described 20. Briefly, peripheral blood mononuclear cells (PBMCs) were isolated from fresh blood drawn in EDTA tubes by standard Percoll gradient centrifugation. The obtained PBMCs were washed twice after centrifugation at 500 × g for 5 min. Macrophages were obtained from differentiated PBMCs, stimulated with 50 ng/mL rhM-CSF (PeproTech, 30025) in RPMI 1640 medium supplemented with 10% heat-inactivated FBS, and incubated in a 5% CO_2_ atmosphere at 37 °C for 7 days. The purity of CD14^+^ cells was verified by flow cytometry. CD14^+^ cells with a purity greater than 90% were used in the experiment. CD14^+^ macrophages were treated with 10, 20, and 30 mM LA (Sigma, L6402) for 3 h or TCM at 48 h in a 21% O_2_ atmosphere at 37 °C for 24 h.

### Flow cytometry

The phenotype of the macrophages was detected by incubation with the appropriate fluorochrome-conjugated monoclonal antibodies (mAbs) for 30 min at 4 °C for cell surface staining. The following human mAbs specific for surface antigens were used: FITC-conjugated anti-human CD14 (61D3) antibody (eBioscience, 85-11-0049-42), PE-Cyanine7-conjugated anti-human CD86 (IT2.2) antibody (eBioscience, 85-25-0869-42), PE-Cyanine7-conjugated anti-human CD206 (19.2) antibody (eBioscience, 85-25-2069-42). All antibodies. Flow cytometric analysis was performed on an LSR II flow cytometer (BD Biosciences, San Jose, CA, USA). Data were analyzed with FlowJo version 10.0.7 software (TreeStar, Asland, OR, USA).

### RNA extraction and reverse transcription

Total RNA was extracted from 5 × 10^5^ cells in each well of a 12-well plate after treatment with or without 20 mM LA for 48 h using TRIzol (Tiangen Biotech, DP424) according to the manufacturer's instructions and assessed for quality and quantity using absorption measurements with a Nanodrop 2000 (Thermo, San Diego, CA, USA). Reverse transcription of 1000 ng of RNA was performed according to the manufacturer's instructions (Takara, RR047A).

### Real-time quantitative polymerase chain reaction (RT-qPCR)

Gene expression analysis was performed using SYBR Green Dye (Takara, RR820A) in Q225 (Kubo Biotech, Beijing, China) according to the manufacturer's instructions. Gene-relative expression was calculated using the 2^-ΔΔCT^ method and normalized to a reference control (ACTB). Primers for RT-qPCR were designed with the assistance of online tools (Primer design on NCBI) using at least one exon junction binding site per primer pair where possible. A complete list of primers used is available in the supporting information, as shown in Table S1.

### Western blotting

Cells were harvested, and total protein was extracted with ice-cold RIPA lysis buffer (Beyotime, P0013B) containing a protease inhibitor cocktail (Servicebio, G2006). The cell suspension was sonicated on ice (three cycles for 5 s with continuous pulsation) and centrifuged at 12,000 × g for 15 min at 4 °C. Then, the supernatant was collected, and the protein concentration was measured using a BCA protein assay kit (Biosharp, P0010S). Samples containing 40 μg of protein were separated by 10%-12% SDS-PAGE and transferred onto a 0.22-μm polyvinylidene difluoride membrane (Bio-Rad, 1620177). The membranes were blocked with 5% fat-free milk (BD, BD232100) for 30 min at room temperature. Then, the cells were incubated overnight at 4 °C with primary antibodies: anti-NDUFA1 antibody (Abclonal, A8326), anti-SDHB antibody (Abclonal, A10821), anti-UQCRC2 antibody (Abclonal, A3339), anti-COX4L2 antibody (Abclonal, A18354), anti-anti-ATPF1 antibody (Abclonal, A5099), anti-HIF-1α antibody (ProteinTech, 66730-1-Ig), anti-HIF-2α antibody (Abclonal, A7553), anti-SRC antibody (Abclonal, RK06002), anti-p-SRC (Y416) antibody (Abclonal, RK06002), anti-p-LDHA(Y10) antibody (ImmunoWay, YP1385), anti-p-LDHA (Tyr239) (Affinity, AF7173), anti-INOS antibody (Abclonal, A0312), anti-Arginase 1 antibody (Abclonal, A1847), anti-VEGF antibody (Abclonal, A12303) and anti-β-actin antibody (Abclonal, AC038). Subsequently with the corresponding secondary antibody conjugated to horseradish peroxidase for 1 h at room temperature. The immunoreactive bands were detected with an ECL solution (Servicebio, G2014) using Image Lab software (Bio-Rad). The relative band density was determined using ImageJ2x software (National Institutes of Health).

### Reactive oxygen species (ROS) measurement

The intracellular ROS content was measured by commercial assay following the manufacturer's instructions. Briefly, the medium was aspirated. Then, the cells were washed with HBSS and incubated with HBSS containing 1 μM DCFH-DA (Nanjing Jiancheng Bioengineering Institute-E004-1-1) for 30 min at 37 °C in 5% CO_2_. ROS generation was observed under a fluorescence microscope (Olympus, Japan) at 200 × magnification. The intensity of the fluorescence was determined using ImageJ2x software (National Institutes of Health).

### Extracellular and intracellular pH measurement

The extracellular pH in the culture medium was measured with an MP220 Basic pH/mV meter (Mettler Toledo). The intracellular pH was measured by commercial assay following the manufacturer's instructions. Briefly, the cells were washed with HBSS and incubated with HBSS containing 5 μM BCECF AM (Thermo Fisher, B1150) for 30 min at 37 °C in 5% CO_2_. Then, the cells were washed with HBSS. Intracellular pH was observed with a Leica TCS SP5 Laser Scanning Confocal Microscope Running Leica LAS-AF software. A dual-excitation ratio with λ1 = 490 nm, λ2 = 440 nm and fixed emission at 535 nm was used for the detection of BCECF and AM fluorescence.

### NAD^+^ / NADH ratio measurement

Intracellular NAD^+^ and NADH were measured by a commercial assay kit (Sigma, MAK037-1KT) following the manufacturer's instructions. Briefly, the medium was aspirated; the cells were washed with PBS and lysed with extraction buffer and centrifuged at 12,000 × g at 4 °C for 10 min. The supernatant was used for NADt (total NAD^+^ and NADH) or NADH determination after decomposing NAD^+^ at 60 °C for 30 min. Then, alcohol dehydrogenase working solution was added to the reaction and incubated at 37 °C for 10 min. The optical density was read at 450 nm after incubation with 10 μL of detection buffer at 37 °C for 30 min. The NAD^+^/NADH ratio was determined from the measured NADt and NADH values by the following formula: (NADt-NADH)/NADH.

### Statistical analyses

All data were analyzed using GraphPad Prism 7 statistical software (La Jolla, CA). Differences between two groups were analyzed using Student's *t* test, and multigroup comparisons were assessed by one-way ANOVA. The data are presented as the mean ± SEM. Differences were considered statistically significant if the *P* value was less than 0.05. Significance is indicated as follows: *P < 0.05, **P < 0.01, ***P < 0.001.

## Results

### Decidual macrophages exhibit the M2 phenotype in NP but the M1 phenotype in RPL

**Figure 1:**
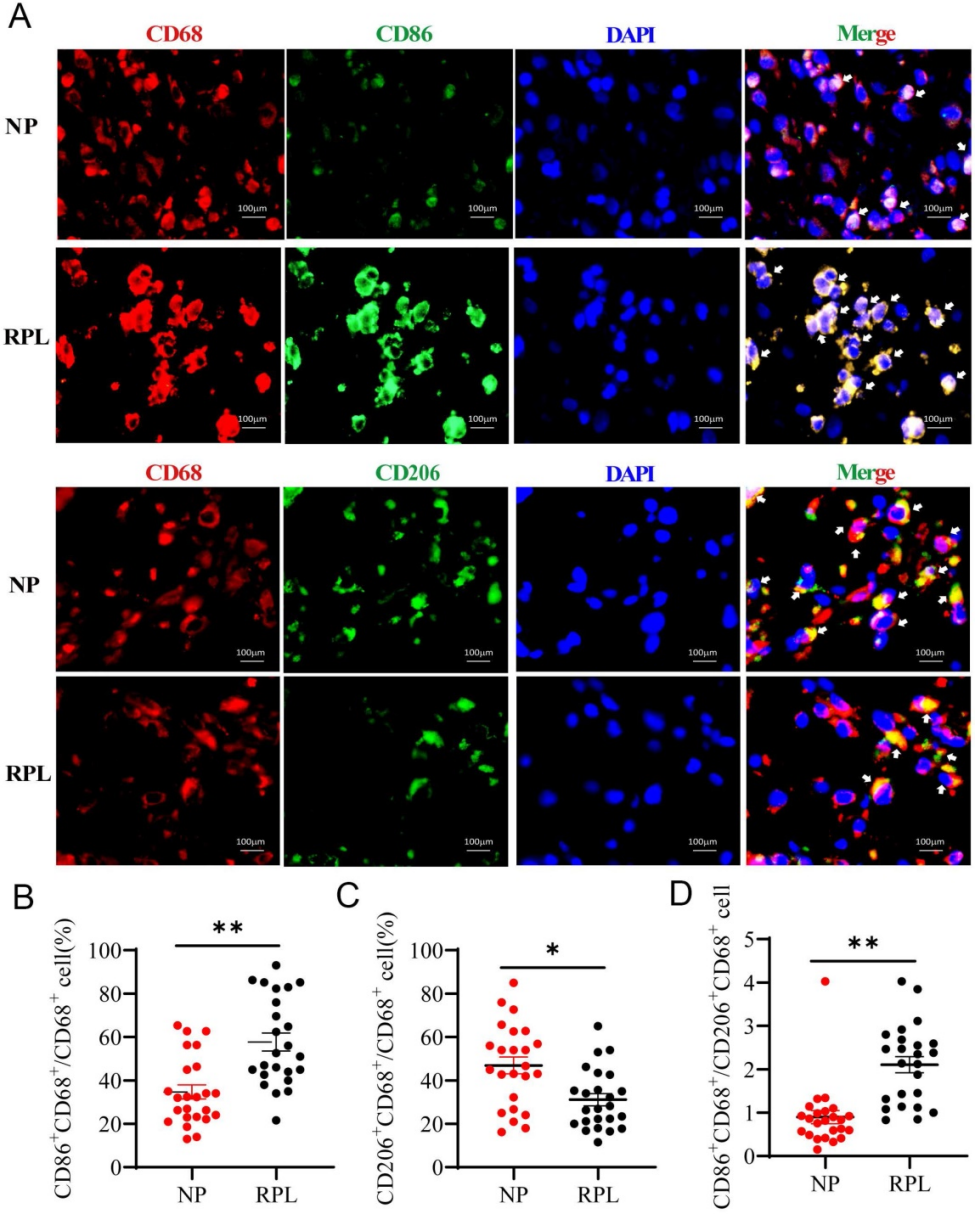
** Decidual macrophages polarized to the M2 phenotype in NP but to the M1 phenotype in RPL patients. (A)** Representative staining of decidual macrophages by immunofluorescence in women with NP (n = 24) and RPL (n = 24). **(B, C)** Quantification of M1 (CD86^+^ CD68^+^/CD68^+^) and M2 (CD206^+^ CD68^+^/CD68^+^) macrophages was performed by Image-Pro Plus 6.0. **(D)** The ratio of M1 / M2 macrophages (CD86^+^ CD68^+^/CD206^+^ CD68^+^) was calculated based on the quantification of M1 and M2 macrophages. Data are shown as the means ± SEM. **p < 0.01, ****p < 0.0001.

Macrophages are tissue-residual immune cells, and their phenotype and functions are regulated by different microenvironments 3. Pregnancy-associated macrophages are considered different from typical macrophages 1. To confirm the characteristics of pregnancy-associated macrophages in women with NP and RPL, the proportion of M1 (CD86^+^ CD68^+^/CD68^+^) and M2 (CD206^+^ CD68^+^/CD68^+^) macrophages was measured by Immunofluorescence staining. The results showed that the proportion of decidual M1 macrophages was significantly increased (*p* < 0.0001) (Fig. 1A, B), while that of M2 macrophages was decreased in RPL compared to NP (*p* < 0.001) (Fig. 1A, C). Moreover, the CD86^+^ CD68^+^/CD206^+^ CD68^+^ (M1/M2) ratio increased significantly in RPL (*p* < 0.0001) (Fig. 1D). These findings indicated that decidual macrophages preferentially exhibit the M2 phenotype in NP but the M1 phenotype in RPL.

### Enhanced LA production, anaerobic glycolysis level and HIF-1α abundance in the decidua from RPL patients

**Figure 2:**
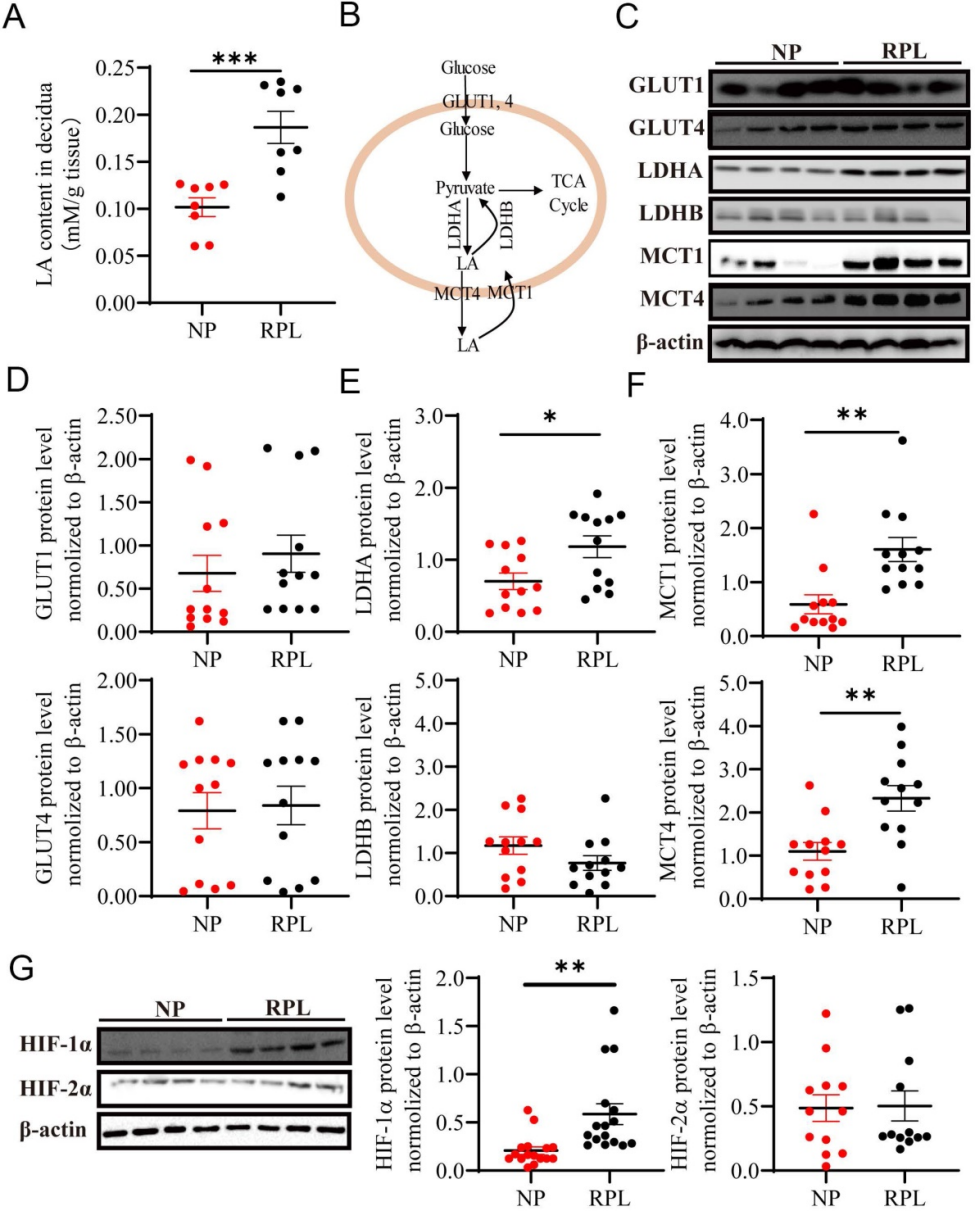
** Enhanced LA production through anaerobic glycolysis in the decidua of RPL patients. (A)** LA levels in the decidua of women with NP (n = 8) and RPL (n =8) were determined by the colorimetric method and normalized to tissue wet weight (mM/g tissue). **(B)** Schematic diagram of glycolysis (LA synthesis and secretion). **(C-F)** The expression and quantification of glycolysis-related proteins (GLUT1, GLUT4, LDHA, LDHB, MCT-1 and MCT-4) (n = 12). **(G)** Hypoxia-marked proteins (HIF-1α and HIF-2α) in the decidua of women with NP and RPL were detected by western blotting (n = 12 - 16). Data are shown as the means ± SEM. *p < 0.05, **p < 0.01, ***p < 0.001. LA, lactic acid; NP, normal pregnancy; RPL, recurrent pregnancy loss.

To decipher the underlying mechanism, we hypothesized that decidual LA and hypoxia may mediate macrophage polarization in the decidua from women with NP and RPL. To address this, first, we examined the abundance of LA in the decidua of women with NP and RPL. Decidual tissues were freshly isolated from women with RPL and gestational age-matched normal pregnancy and analyzed for LA abundance. As shown in Fig. 2A, the LA concentration was significantly increased in the decidua of RPL patients compared to NP women (*p* < 0.001). To determine whether increased LA is associated with dysregulation of LA metabolism (Fig. 2B), we assessed the expression of glycolysis-related proteins that are involved in LA synthesis (i.e. LDHA and LDHB), uptake (i.e. MCT-1) and secretion (i.e. MCT-4) as well as glucose uptake (i.e. GLUT1 and GLUT4) in the decidua from women with RPL vs. NP. Western blotting analysis revealed a remarkable upregulation of LDHA (*p* = 0.019), MCT-1 (*p* < 0.01) and MCT-4 (*p* < 0.01) in the decidua from women with RPL vs. NP (Fig. 2C, E, F). To evaluate oxygen environment in the decidua, key regulators, HIF-1α and HIF-2α, were analyzed and compared for their abundance between NP and RPL. We found that the decidua from RPL patients contained significantly higher levels of HIF-1α compared to NP women (*p* < 0.01) (Fig. 2G). Although RPL decidua exhibited slight increases in GLUT1, LDHB and HIF-2α as well as a slight decrease in GLUT4, no statistically significant difference was observed between the two groups (*p* = 0.456, *p* = 0.142, *p* = 0.914, and *p* = 0.8424, respectively) (Fig. 2C, D). These results suggested that enhanced LA microenvironment was caused by increase in LA production through anaerobic glycolysis in the decidua from RPL patients, and the decidua in RPL entailed a more hypoxic microenvironment compared to NP.

### Increased LA is derived from decidual trophoblasts from RPL patients

**Figure 3:**
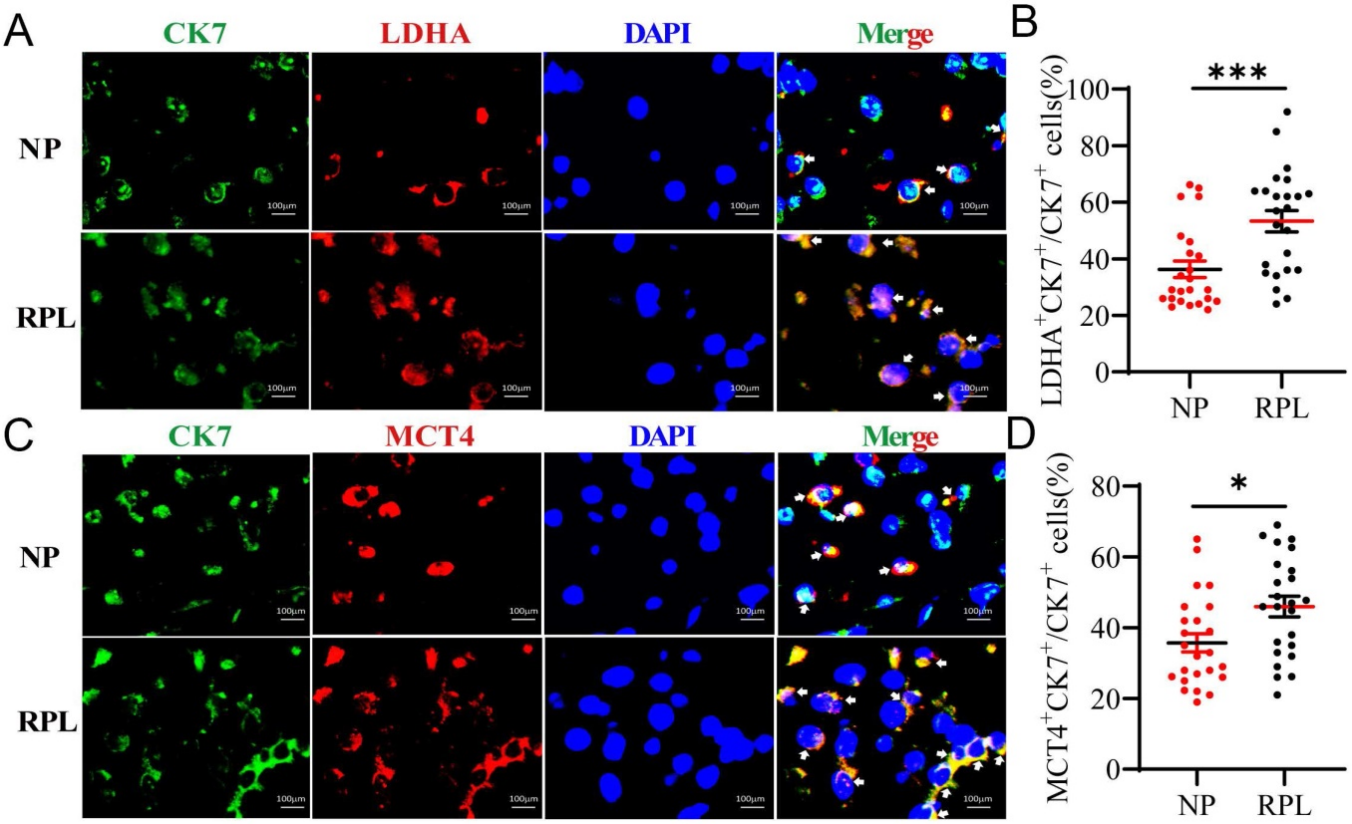
** Trophoblasts contribute to the high level of LA in the decidua of RPL patients. (A, B)** Colocalization and quantification of CK7^+^ (trophoblast marker) LDHA^+^ (LA synthesis) cells in the decidua of women with NP (n = 24) and RPL (n = 24) by immunofluorescence assay. **(C, D)** Colocalization and quantification of CK7^+^ MCT-4^+^ (LA secretion) cells in the decidua of women with NP (n = 24) and RPL (n = 24) by immunofluorescence assay. Data are shown as the means ± SEM. *p < 0.05, ***p < 0.001.

Trophoblasts are important cellular players of embryo implantation. A study showed that LA in the decidua may be synthesized and secreted by embryo-derived trophoblasts 21. To determine the source of decidual LA, we determined whether LDHA and MCT-4 were present in trophoblasts (CK7^+^) in the decidua using immunofluorescence (IF) staining. Colocalization analysis revealed that the proportion of LDHA^+^ CK7^+^ (*p* < 0.001) and MCT-4^+^ CK7^+^ (*p* = 0.012) cells were obviously increased in the decidua from women with RPL compared to the women with NP (Fig. 3A-D). These observations indicated that increased LA was mainly produced in trophoblasts in the decidua, and trophoblasts in RPL decidua produced a higher level of LA than those in NP decidua.

### Trophoblast-derived LA regulates macrophage polarization under normoxia and hypoxia

**Figure 4:**
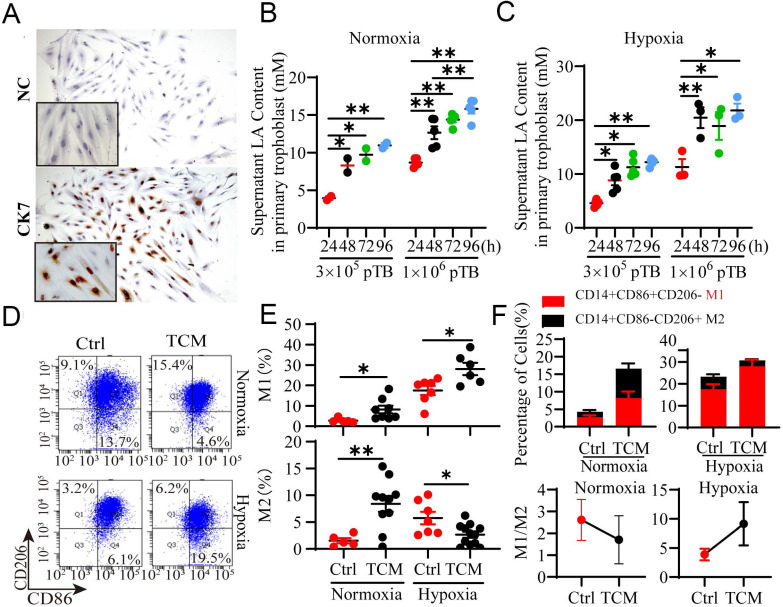
** Trophoblast-derived LA regulates the polarization of PBMC-derived macrophages under normoxia and hypoxia. (A)** Isolated primary trophoblasts were identified via positive staining with CK7 by immunocytochemistry. **(B, C)** The LA level in the supernatant of primary trophoblasts was detected by the colorimetric method after the trophoblasts were cultured in 6 - well plates at a density of 3 × 10^5^ cells or 1 × 10^6^ cells/well in a 21% O_2_ or 1% O_2_ atmosphere for 24h -96 h, respectively (n = 3-6). **(D)** Representative flow cytometry data of M1 (CD14^+^ CD86^+^ CD206^-^) and M2 (CD14^+^ CD86^-^ CD206^+^) macrophages treated with or without TCM under normoxic and hypoxic conditions. **(E)** Statistical analysis of the percentages of M1 (CD14^+^ CD86^+^ CD206^-^) and M2 (CD14^+^ CD86^-^ CD206^+^) macrophages with or without TCM treatment under normoxia and hypoxia (n = 6 - 10).** (F)** The M1/M2 ratio and the percentages of macrophages treated with or without TCM under normoxic and hypoxic conditions. Data are shown as the mean ± SEM. *p < 0.05, **p < 0.01. NC: negative control; TCM: trophoblast conditioned medium.

To further confirm whether trophoblasts can artificially release LA, we isolated and cultured primary trophoblasts (pTB) from the villi of NP women (Fig. 4A, Fig. S1A, B). pTB was exposed to hypoxia condition (1% O_2_) or normoxia condition (21% O_2_) as a control. The results showed that trophoblasts intrinsically secreted LA in a density-dependent (3 × 10^5^ to 1 × 10^6^) and time-dependent (24 h to 72 h) manners (Fig. 4B, C). Thus, hypoxia exposure significantly increased the LA levels in the culture medium from pTB relative to normoxic treatment. Based on the above results and other studies on the modulatory effect of LA on macrophages 7, 22, we hypothesized that trophoblast-derived LA may regulate pregnancy-associated macrophage polarization, and when coupled with hypoxia, may cause macrophages to polarize to the M1 phenotype as observed in RPL patients. To address this, we cultured peripheral monocyte-derived macrophages and compared the polarization pattern of macrophages treated with TCM under normoxic or hypoxic conditions (Fig. 4D). Flow cytometry analysis demonstrated that TCM treatment mainly increased M2 (*p* < 0.01, and *p* = 0.015, respectively) and to less extent, M1 percentage (*p* = 0.044, and *p* = 0.016, respectively), thereby decreasing M1/M2 ratio under normoxia condition (Fig. 4E, F, Fig. S2). This suggested that TCM preferentially promoted macrophage polarization toward M2 phenotype under normoxia conditions. In contrast, under hypoxia condition, TCM elevated M1 macrophage percentage but reduced M2 percentage compared to the control. Apparently, TCM treatment dramatically increased M1/M2 ratio in the context of hypoxia condition (Fig. 4B-E), implying that hypoxic conditions can reverse TCM-induced M2 macrophages polarization. Together, these results indicated that macrophages were biased to M2 subtype under normoxia but to M1 subtype under hypoxia in response to TCM treatment *in vitro*.

### Exogenous LA polarizes macrophages to M2 subtype under normoxia but to M1 subtype under hypoxia through HIF-1α

**Figure 5:**
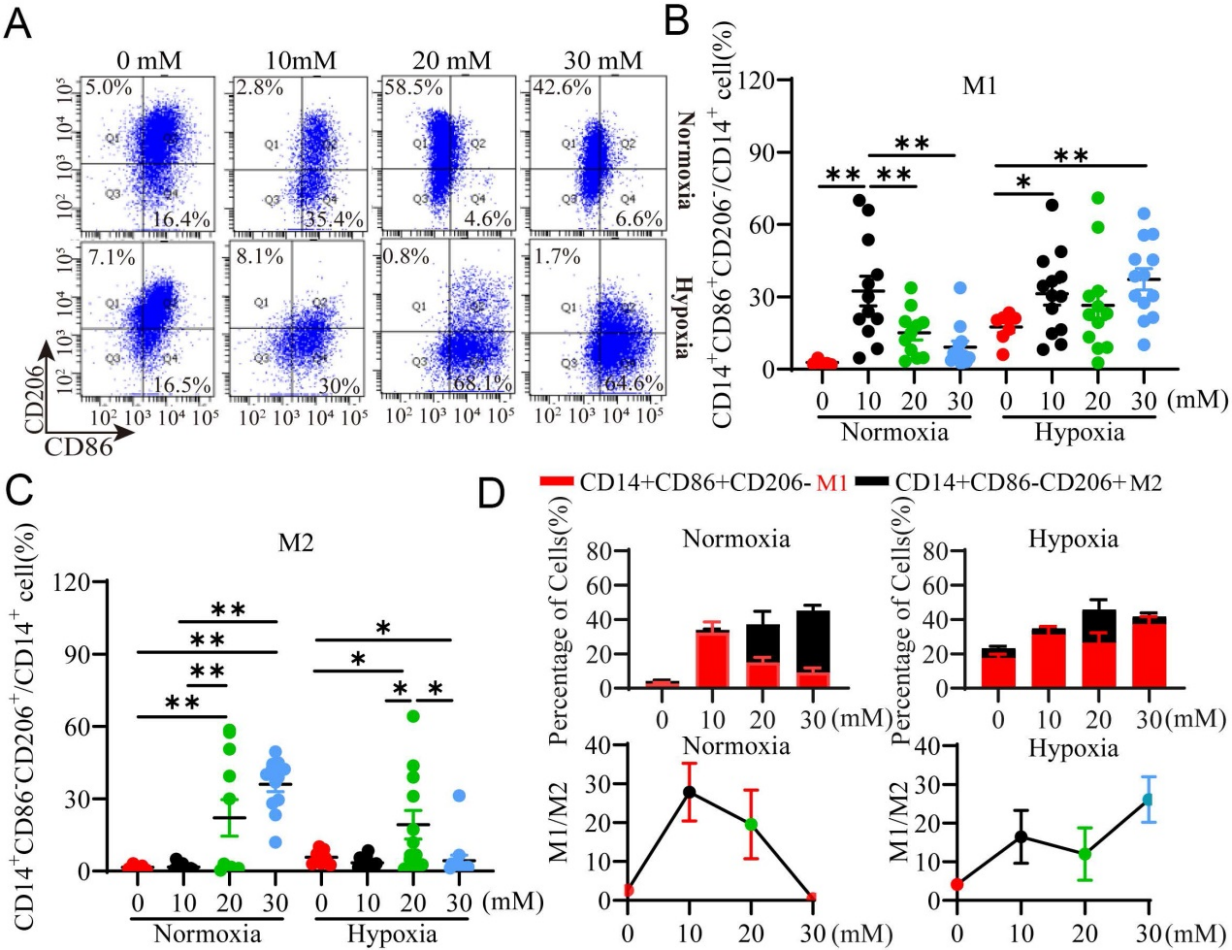
** Exogenous LA regulates the polarization of PBMC-derived macrophages under normoxia and hypoxia. (A)** Representative data of M1 (CD14^+^ CD86^+^ CD206^-^) and M2 (CD14^+^ CD86^-^ CD206^+^) macrophages treated with 1 μM HCl (control as 0 mM LA) or LA (10 mM, 20 mM and 30 mM) for 3 h under normoxic and hypoxic conditions by flow cytometry. **(B, C)** Statistical analysis of the percentages of M1 (CD14^+^CD86^+^CD206^-^) and M2 (CD14^+^CD86^-^CD206^+^) macrophages treated with or without LA under normoxic and hypoxic conditions (n = 7-15). **(D)** The percentages of M1 (CD14^+^ CD86^+^ CD206^-^) and M2 (CD14^+^ CD86^-^ CD206^+^) macrophages (n = 7-15) and M1/M2 ratio. Data are shown as the mean ± SEM. *p < 0.05, **p < 0.01.

To validate the results observed above, we evaluated whether exogenous LA could recapitulate the effect of TCM on macrophage polarization under normoxia and hypoxia. Macrophages were treated with exogenous LA at various concentrations and analyzed for their polarization using flow cytometry. Our results showed that exogenous LA especially at 20 mM and 30 mM mainly increased the percentage of M2 macrophages (All *p* <0.001) relative to M1 macrophages under normoxia (Fig. 5A, B), but predominantly elevated the percentage of M1 subtype versus M2 subtype under hypoxia (Fig. 5A, C). Therefore, the M1/M2 ratio was decreased under normoxia but increased under hypoxia in the presence of exogenous LA especially at more than 20 mM. This phenomenon is similar as what we observed in women with NP and RPL, respectively. Altogether, these results indicated that macrophages were preferentially biased toward the M2 phenotype under normoxia but toward the M1 phenotype under hypoxia in response to exogenous LA treatment, which supported the effect of TCM (containing trophoblast-derived LA) on macrophage polarization under normoxia or hypoxia.

**Figure 6:**
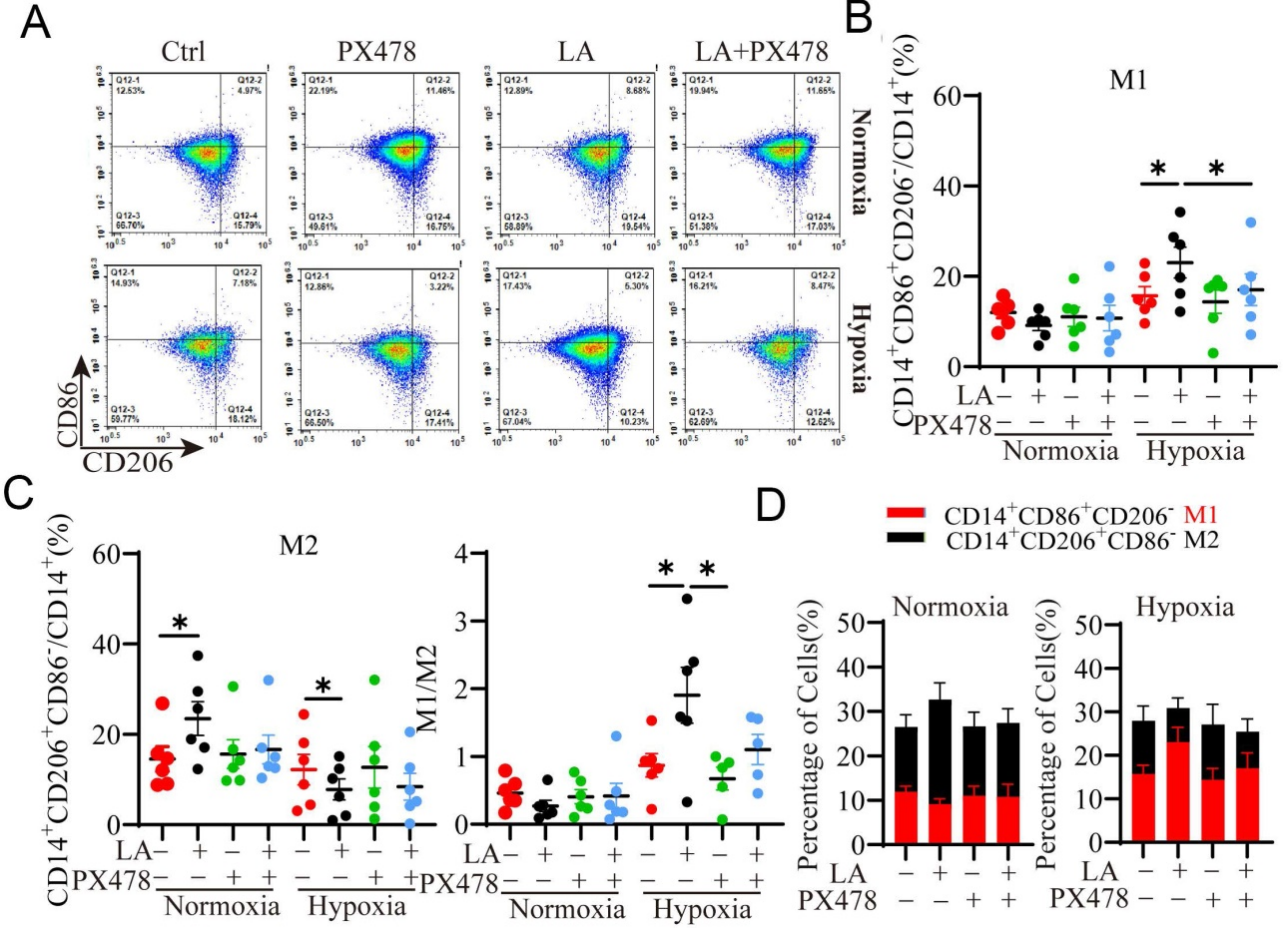
** Blockade of HIF-1**α** inhibits PBMC-derived macrophage polarization regulated by exogenous LA (20 mM) under normoxia and hypoxia. (A)** Representative flow cytometry data of M1 (CD14^+^ CD86^+^ CD206^-^) and M2 (CD14^+^ CD86^-^ CD206^+^) macrophages treated with or without LA under normoxic and hypoxic conditions. **(B, C)** The statistical data of the percentages of M1 (CD14^+^ CD86^+^ CD206^-^) and M2 (CD14^+^ CD86^-^ CD206^+^) macrophages treated with or without LA in the presence or absence of PX478 (20 µM, HIF-1α inhibitor) under normoxic and hypoxic conditions (n = 6). **(D, E)** The M1/M2 ratio and the percentages of M1 and M2 macrophages (n = 6). Data are shown as the mean ± SEM. *p < 0.05.

HIF-1α, a master regulator of cellular response to oxygen levels plays an important role in regulating glycolytic metabolism and OXPHOS 20, 23. It has been shown that LA promoted LA transport and glycolysis/OXPHOS interconversion in glioblastoma 24. Next, we investigated whether HIF-1α was implicated in LA-mediated macrophage polarization under normoxia or hypoxia. To this end, macrophages were treated with 20 mM LA in the presence or absence of HIF-1α inhibitor, PX478 under normoxia and hypoxia conditions (Fig. 6). As expected, PX478 did not affect LA-promoted M2-subtype polarization of macrophages under normoxia (*p* = 0.431) (Fig. 6A, C). In contrast, under hypoxia, inhibition of HIF-1α attenuated LA-induced M1-subtype polarization (*p*= 0.021) (Fig. 6A, B). In addition, the M1/M2 ratio was decreased under hypoxia (*p* = 0.036) (Fig. 6D, E). These results indicated that LA-induced macrophage polarization under hypoxia was mediated by HIF-1α.

### LA regulates metabolic reprogramming during macrophage polarization under normoxic and hypoxic conditions

**Figure 7:**
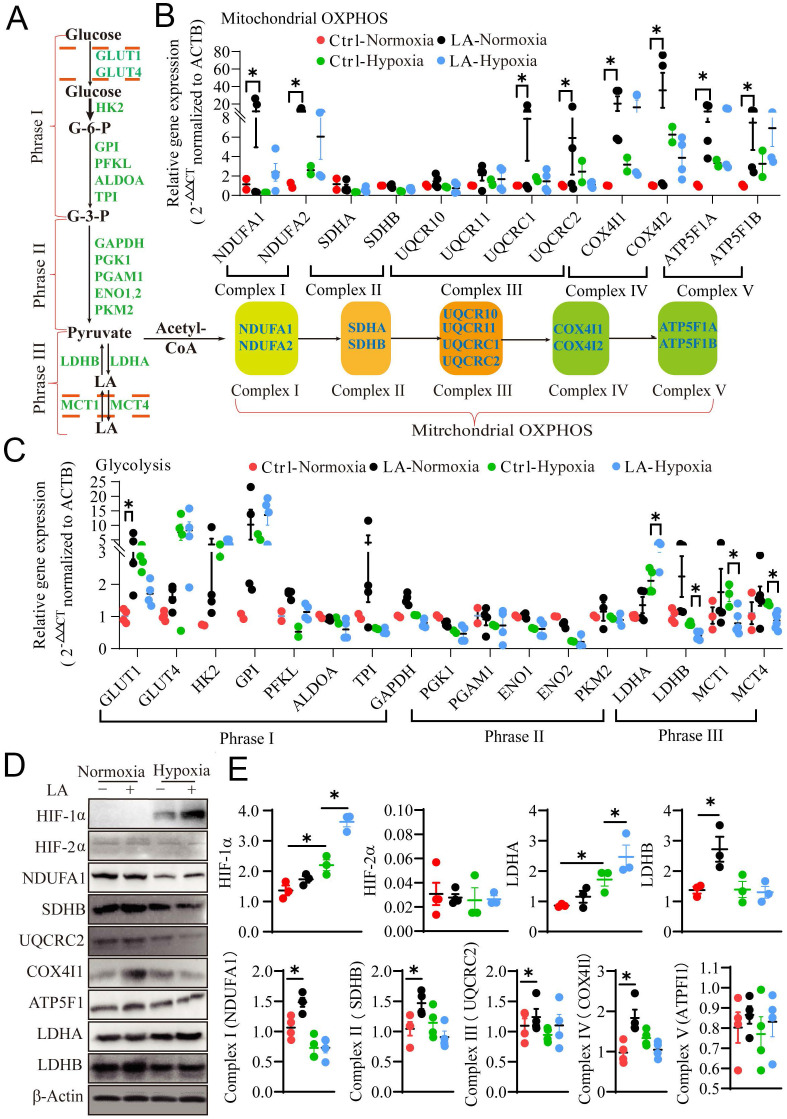
** Exogenous 20mM LA regulates PBMC-derived macrophage polarization through metabolic reprogramming under normoxia and hypoxia. (A)** Schematic diagram of mitochondrial OXPHOS and glycolysis. **(B, C)** The mRNA expression of mitochondrial OXPHOS- and glycolysis-related genes in macrophages treated with or without LA (20 mM) under normoxia and hypoxia (n = 3-6). **(D, E)** Quantification of protein expression participating in mitochondrial OXPHOS (NDUFA1, SDHB, UQCRC2, Cox4I1 and ATP5F1), hypoxia (HIF-1α and HIF-2α) and glycolysis (LDHA and LDHB) regulation in macrophages treated with or without LA (20 mM) under normoxia and hypoxia by western blotting (n = 3-6). Data are shown as the mean ± SEM. *p < 0.05. OXPHOS, Oxidative phosphorylation.

Prior studies have demonstrated that metabolic reprogramming of glycolysis and mitochondrial OXPHOS switching in macrophages mediates their differentiation and polarization in cancer tissues 24, 25. Here the question arises as to whether LA can affect metabolic reprogramming in different oxygen environment, leading to macrophage polarization. To address this question, we interrogated the effect of exogenous LA on glycolysis and mitochondrial OXPHOS by examining the mRNA and protein expression of genes associated with glycolysis, including glucose uptake, pyruvate synthesis and LA metabolism, as well as mitochondrial OXPHOS in exogenous LA-treated macrophages under normoxia or hypoxia (Fig. 7A). Our results showed that under normoxia, exogenous LA (20 mM) promoted OXPHOS in macrophages as evidenced by increases in mRNA expression levels of NADH dehydrogenase [ubiquinone] 1 alpha subcomplex subunits (NDUFA1 & 2), cytochrome b-c1 complex subunits (UQCRC1 & 2), cytochrome c oxidase subunit 4 isoform (COX4I1 & 2) and ATP synthase F1 subunit (ATP5F1A & B) (*p* = 0.041, *p* = 0.036, *p* = 0.029, *p* = 0.042, *p* = 0.047, *p* = 0.026, *p* = 0.032, and *p* = 0.031, respectively) (Fig. 7B). Nevertheless, under hypoxia, exogenous LA (20 mM) promoted glycolysis in macrophages as evidenced by upregulation of LDHA and downregulation of LDHB mRNA (*p* = 0.049, *p* = 0.042, *p* = 0.044, *p* = 0.043, and *p* = 0.048, respectively) (Fig. 7C). Moreover, western blotting results revealed that LA promoted the protein expression of HIF-1α and LDHA under hypoxia (*p* = 0.012, and *p* = 0.034, respectively), but increased the abundance of SDHB, COX4I1 and LDHB proteins under normoxic conditions (*p* = 0.042, *p* = 0.036 and *p* = 0.031, respectively). Statistical analysis showed no significant changes in the content of HIF-2α and ATP5F1 proteins under either normoxia or hypoxia (*p* = 0.757, and *p* = 0.545, respectively) (Fig. 7D, E). In addition, hypoxia alone remarkably enhanced the abundance of HIF-1α and LDHA proteins (*p* = 0.035, and *p* = 0.028, respectively) but attenuated the expression of NDUFA1 and UQCRC2 proteins (*p* = 0.032, and *p* = 0.029, respectively) (Fig. 7D, E). Otherwise, the same effect of LA (20 mM) on HIF-1α expression was observed under normoxia (21% O_2_) and physical normoxia (5% O_2_) (Fig. S3A, B). Taken together, these results uncovered that treatment with LA, hypoxia or both LA and hypoxia distinctly influenced metabolic reprogramming in macrophages, which may lead to alterations in macrophage polarization.

### Exogenous LA regulates macrophage polarization through SRC / LDHA pathway

**Figure 8:**
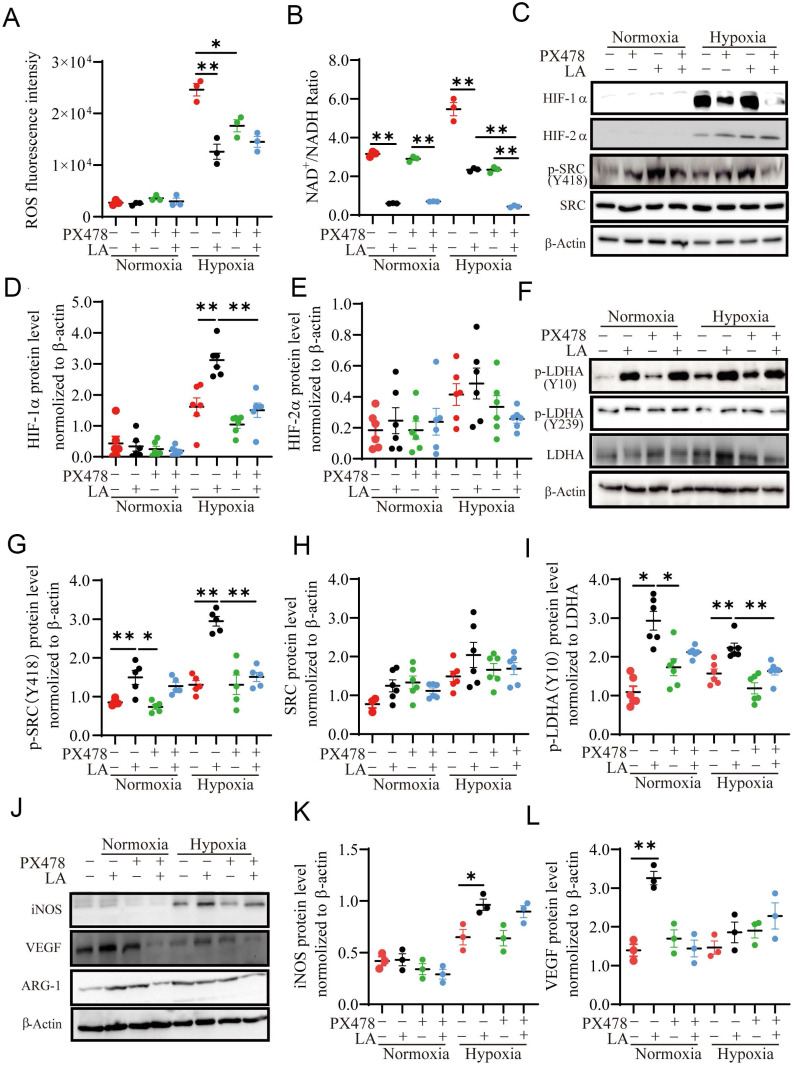
** Exogenous LA (20 mM) regulates PBMC-derived macrophage polarization through the HIF1-α/SRC/LDHA pathway. (A)** ROS fluorescence intensity (n=3). **(B)** NAD^+^/NADH ratio (n = 3). **(C-L)** Representative data and quantification of HIF-1α, HIF-2α, p-SRC (Y418), SRC, p-LDHA (Y10), p-LDHA (Y239), LDHA, INOS, VEGF and ARG1 protein expression in macrophages treated with or without 20mM LA in the presence or absence of PX478 under normoxia and hypoxia (n = 3-6). Data are shown as the mean ± SEM. *p < 0.05, **p < 0.01.

Redox imbalance and microenvironment acidification induced by hypoxia and glycolysis have been associated with macrophage polarization 26, 27. It is recognized that ROS is reduced by hypoxia 28, but the regulation of ROS by lactic acid is controversial at present 29, 30. Some study showed that LA can fuel mitochondrial ROS generation and cause extracellular acidification 27, 31. To investigate whether LA can induce redox imbalance and microenvironment acidification in macrophages, we assessed the ROS content, extracellular and intracellular pH value in macrophages treated with 20 mM LA. Our results showed that either normoxia alone or LA did not alter ROS production under normoxia (*p* = 0.461) (Fig. 8A, Fig. S4A). Hypoxia alone dramatically enhanced the levels of NAD^+^/NADH ratio and ROS (Fig. 8A, Fig. S4D, E), which were blocked by PX478. LA reduced the levels of NAD^+^/NADH ratio and ROS production in macrophages under hypoxia. This suggested that LA-induced reduction of ROS and NAD^+^/NADH ratio might due to its upregulation of HIF-1α in macrophages. Although hypoxia alone or with LA slightly decreased intracellular and extracellular pH, the difference was not statistically significant as compared with respective controls (hypoxia alone vs. normoxia alone: *p* = 0.5121, *p* = 0.0981) (Fig. S4B, C). Interestingly, blockage of HIF-1α augmented LA-induced decrease in NAD^+^/NADH ratio under hypoxia (*p* < 0.01). In support of this, LA highly increased the abundance of HIF-1α but not HIF-2α in hypoxia-treated macrophages (*p* < 0.01, and *p* = 0. 421, respectively) (Fig. 8C-E).

To evaluate whether LA affects p-SRC/LDHA pathway, we examined the abundance of SRC, p-SRC, p-LDHA (Y10/Y239) and LDHA proteins in LA-treated macrophages under normoxia (21% O_2_), hypoxia (1% O_2_), and physical normoxia (5% O_2_) (Fig. S3A). Our results showed that LA (20 mM) upregulated the protein expression of p-SRC (Y418) and p-LDHA (Y10) under normoxia (*p* < 0.01, and *p* = 0.032, respectively) (Fig. 8C, F, I, Fig. S3A), and LA-induced increase in p-SRC and p-LDHA expression was augmented under hypoxia (both *p* < 0.01). However, LA (20 mM) did not change the contents of SRC (*p* = 0.856, and *p* = 0.753, respectively) (Fig. 8H) and p-LDHA (Y239) (*p* = 0.242, and *p* = 0.493, respectively) under normoxia or hypoxia (Fig. S5B). Moreover, blocking HIF-1α with PX478 could not inhibit LA-induced upregulation of p-SRC (Y418) and p-LDHA (Y10) under normoxia (*p* = 0.786, and *p* = 0.851, respectively), PX478 attenuates the abundance of these two proteins in macrophages under hypoxia (both *p* < 0.01) (Fig. 8G, I). This suggested that HIF-1α mediates LA-induced increase of p-SRC and p-LDHA proteins in macrophages exposed to hypoxia not normoxia (Fig. 8C, F, G, J).

Furthermore, LA (20 mM) did not significantly change iNOS content (*p* = 0.865), instead remarkably upregulated VEGF and ARG-1 protein expression under normoxia (*p* = 0.042, and *p* = 0.038, respectively), which was inhibited by PX478 (Fig. 8J, K, L). This suggested that LA-induced upregulation of VEGF and ARG-1 was modulated through HIF-1α-independent manner under normoxia. On the other hand, LA increased iNOS abundance but did not significantly affect the levels of VEGF and ARG-1 expression under hypoxia (Fig. 8J, K, L).

### Blockade of LA intake with AZD3965 (MCT-1 inhibitor) could rescue pregnancy in the abortion-prone mouse model

**Figure 9:**
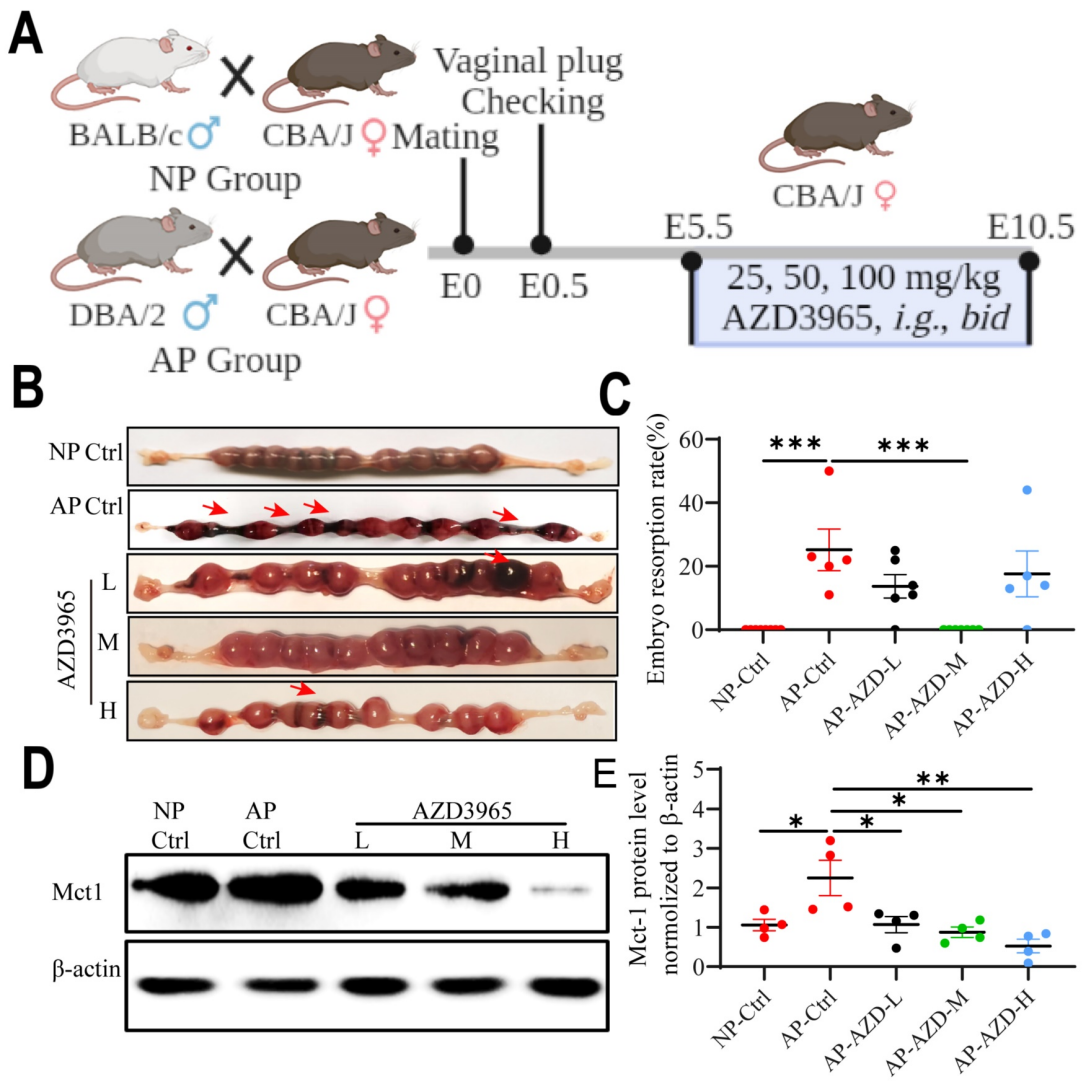
** Blocking LA intake with MCT-1 inhibitor (AZD3965) could rescue pregnancy in abortion-prone mouse model. (A)** Schematic diagram of animal experiment protocol. **(B)** Representative macroscopic view of uterus and fetuses in NP and AP mice with or without AZD3965 treatment at doses of 25 mg/kg, *bid* (L), 50 mg/kg, *bid* (M) and 100 mg/kg, *bid* (H), respectively. **(C)** Embryo resorption rate in NP and AP mice with or without AZD3965 treatment (n = 5-8). **(D-E)** Representative data and quantification of Mct-1 protein expression in uterus of NP and AP mice with or without AZD3965 treatment (n = 4). Data are shown as the mean ± SEM. *p < 0.05, **p < 0.01, ***p < 0.001. L, low; M, moderate; H, high.

To determine whether blockade of LA intake could affect the pregnancy outcomes, NP mice and AP mice model were used for *in vivo* study. The AP mice were orally administrated with different doses of AZD3965 (MCT-1 inhibitor) from E5.5 to E10.5 (Fig. 9A). The results show that the embryo resorption rates were significantly increased in AP control group compared to NP control group (*p* < 0.001) (Fig. 9B, C). AZD3965 at moderate doses could significantly decrease the embryo resorption rates in AP mice (*p* < 0.001) (Fig. 9B, C). Moreover, AZD3965 at low, moderate and high doses could decrease the expression of Mct-1 protein in AP mice (*p* = 0.031, *p* = 0.011, and *p* = 0.002, respectively) (Fig. 9D, E). These results indicated that blockade of LA intake could rescue the pregnancy in AP mouse model and MCT-1 might be a potential intervention target.

## Discussion

LA, as a novel signaling molecule, plays a pivotal role in cell migration, invasion, growth, angiogenesis, and immune tolerance 32. In particular, 'lactate shuttling' through MCT-1/MCT-4 between cancer cells and macrophages leads to macrophage polarization 9. However, little is known about the immunological role of LA at the maternal-fetal interface, specifically in decidual macrophage polarization. In the current study, for the first time, we discovered that LA triggers M2 polarization in decidual macrophages and enhances the expression of genes associated with OXPHOS under normoxia but triggers M1 polarization via enhancing the expression of genes associated with glycolysis under hypoxia. Inhibition of HIF-1α with PX478 reversed the polarization of macrophages. Moreover, decidual macrophages exhibited M2 polarization in pregnant women with NP, but they exhibited M1 polarization in those with RPL. Levels of LA and HIF-1α as well as glycolysis increased in decidua from pregnant women with RPL in comparison with those with NP. Trophoblasts isolated and cultured from the villi of pregnant women artificially secreted LA, especially under hypoxia. The SRC/LDHA pathway underlies the mechanism by which LA triggers pregnancy-associated macrophage polarization, in which VEGF expression was enhanced in a HIF-1α-independent manner under normoxia, but INOS expression was enhanced in a HIF-1α-dependent manner under hypoxia. Finally, blockade of LA intake by AZD3965 (MCT-1 inhibitor) could decrease the embryo resorption rate in AP mouse model. These findings reveal a new phenomenon in which accumulation of LA in decidua of RPL might account for the polarization of decidual macrophages biased to M1 phenotype, which may be relevant to the disruption of immune tolerance in RPL.

Oxygen concentration in uterine is an important regulator of embryo implantation and invasion. The oxygen concentration in normal uterine is maintaining 5% O_2_ as a normoxia environment 33. After implantation and during early pregnancy, the embryo stays in a hypoxia environment (1%-3% O_2_), which is led by spiral artery remodeling with vascular transformation. When the uteroplacental circulation is established, decidual hypoxia gradually relieved at the final stage of early pregnancy, and the oxygen concentration increased to a higher level than the normal uterine (reach to 5%-8% O_2_ at 12 weeks) 33, 34. However, failures in uterine spiral artery remodeling result in poor placental perfusion, endothelial dysfunction, and severe hypoxia, which are regarded as accounting for the etiology of pregnancy-related diseases, including miscarriages in early pregnancy 3. In line with this concept, our results also found that women with RPL showed significant hypoxia (high HIF-1α expression) in the decidua. In addition, the LA level was significantly enhanced, and glycolysis-related proteins, including LDHA, MCT-1 and MCT-4, were highly expressed in the decidua of RPL patients compared to NP women. These results indicated that hypoxia and LA accumulation were obvious in the decidua of RPL patients. As previous studies reported, LA, as an important metabolic molecule for the exchange of information between mother and fetus, has the function of providing energy for embryonic development, promoting endometrial dissociation, and inducing spiral artery remodeling and immune microenvironment homeostasis 8. However, the risk of exaggerated hypoxia and accumulated LA in the uterine microenvironment of RPL patients needs in-depth clarification.

Trophoblasts participate in embryo implantation by regulating maternal-fetal immune tolerance and invasion ability after sensing changes in the uterine microenvironment (cytokines and hypoxia, etc.) 35, 36. *In vitro* studies have shown that trophoblasts exhibit cellular biological characteristics similar to the high proliferation and invasion of tumors 37, 38. For example, in the first trimester, preimplanted blastocysts can invade the endometrium with key metabolic characteristics, namely, glycolysis 8. During proliferation and invasion, tumor cells can synthesize and secrete LA through anaerobic glycolysis 11. Therefore, LA in the decidua of women with NP and RPL may be secreted by trophoblasts. Indeed, the current results in our study showed that the trophoblasts in the decidua of women with RPL highly expressed LDHA (inducing LA synthesis). This finding indicates that the source of exaggerated LA levels in the decidua might come from trophoblasts. In addition, we further proved that trophoblasts could artificially synthesize and secrete LA *in vitro* under normoxia and hypoxia. Under hypoxia, trophoblasts produce more LA than under normoxia. Therefore, trophoblasts seem to exhibit the metabolic characteristics of tumor-like anaerobic and aerobic glycolysis (Warburg effect). Therefore, the high level of trophoblast-derived LA in hypoxia may be related to the occurrence of RPL (lower than the physical normal oxygen concentration 5% O_2_). This finding indicates that the source of exaggerated LA levels in the decidua might come from trophoblasts, which may be related to the occurrence of RPL.

Decidual macrophages exhibiting M1 polarization are an important immune characteristic of RPL. This has been confirmed by numerous studies and our study 2. In this study, we also observed that decidual macrophages in women with RPL showed M1 polarization, while in women with NP showed M2 polarization. Importantly, our study found that the decidua of women with RPL exhibited a high degree of hypoxia and a high LA microenvironment. We speculated that the polarization of decidual macrophages in women with NP and RPL may be related to the LA levels and oxygen environment in the decidua. An appropriate microenvironment including LA levels and oxygen concentration in the decidua is beneficial for early normal pregnancy, while an exaggerated status is detrimental due to the interruption of immune tolerance, similar to what we observed in RPL. Our *in-vitro* studies further approve these clinical findings. What we observed is that PBMC-derived macrophages were preferentially biased to the M2 phenotype under normoxia but to the M1 phenotype under hypoxia in response to either exogenous LA treatment or TCM (containing trophoblast-derived LA). Our *in-vitro* results under normoxia are consistent with the tumors' studies 39, 40. A study in gastric cancer by Zhang *et al*. 39 showed that LA can skew macrophages toward a M2-like phenotype. They treated THP-1 cells or human monocytes with either exogenous LA or gastric cancer cell-derived conditioned media under normoxia, and found significantly increased expression of M2-related markers (IL-10, CD163, and ARG1) and faintly decreased expression of M1-related markers (IL-1, CCR7, and INOS). There are few *in-vitro* studies in tumors using hypoxic condition to investigate the effects of LA or tumor-conditioned media on the macrophage polarization. In a recent study of colon cancer by Martins *et al*. 41, they co-cultured human primary macrophages with colon cancer cells under 20% (normoxia) and 1% (hypoxia) O_2,_ and found that hypoxia drived macrophages into a M1-phenotype. Their results support our findings that normoxia and hypoxia have different effects on the polarization of macrophages.

Macrophages exhibit different metabolic characteristics due to changes in the oxygen environment in tissues 6. Classic M1 macrophages exhibit glycolysis-based metabolic characteristics, while M2 macrophages exhibit OXPHOS 6. HIF-1α is an important transcriptional regulatory molecule for glycolysis and OXPHOS-related proteins 25. Many studies have confirmed that in tumor cells, T cells and macrophages, HIF-1α mediates the expression of multiple proteins involved in glucose metabolism pathways (glycolysis and OXPHOS), such as HK2, LDHs and PDKs 39, 42, 43. In particular, HIF-1α regulates LDHA-dependent LA synthesis and LDHB and mitochondrial-L-lactate dehydrogenase-mediated LA oxidation. Meanwhile, mitochondrial MCT-1 (mMCT-1) promotes the absorption of LA by mitochondria and energy supply mediated by the tricarboxylic acid cycle and is related to the return of protons to the mitochondrial matrix 44. In the current study, we found that exogenous LA promotes glucose uptake (characterized by the upregulation of *Glut1* and *Glut4* mRNA genes) and activation of glucose 3 phosphate-pyruvate conversion (characterized by the upregulation of *Tpi* and *Gapdh* mRNA genes) under normoxic conditions and promotes LA oxidative decomposition (characterized by the upregulation of *Ldhb* mRNA and protein). Therefore, exogenous LA could be metabolized through the mitochondrial tricarboxylic acid cycle and respiratory chain to form ATP. Moreover, our study shows that exogenous LA promotes the expression of mRNAs related to the mitochondrial respiratory chain complex of macrophages under normoxic conditions, including NDUFA1 and NDUFA2 (complex I), SDHB (complex II), UQCRC1 and UQCRC2 (complex III), COX4I1 and COX4I2 (complex IV), and ATP5A1 and ATP5A2 (complex V). Under hypoxia, exogenous LA promotes the mRNA expression of the LDHA gene and inhibits that of the GLUT, MCT-1 and MCT-4 genes in macrophages. Correspondingly, exogenous LA inhibits the expression of SDHB and COX4I1 proteins in macrophages under hypoxia. Therefore, LA blocks the glucose-based energy supply pathway and converts glucose metabolism to an energy supply mode based on LA oxidative metabolism.

HIF-1α and HIF-2α are important oxygen response element proteins in cells 32. Both are involved in the regulation of glucose metabolism, redox imbalance and the polarization of tumor-associated macrophages 10, 39. It has been confirmed that LA could promote M2 polarization by upregulating HIF-2α and HIF-1α in Lewis lung carcinoma and gastric cancer, respectively 10, 39. However, we did not find that exogenous LA influenced HIF-2α expression on pregnancy-associated macrophages under normoxia and hypoxia. Instead, it upregulates HIF-1α expression under hypoxic conditions. This may be due to HIF-1α, but not HIF-2α, being present in the endometrium during the menstrual cycle and early pregnancy 45. Moreover, LA reduced ROS production and the NAD^+^/NADH ratio in macrophages. It may due to the upregulated HIF-1α and fuel the bioenergetics of macrophage mitochondria, even OXPHOS 28, 46, 47. In addition, we observed that the regulation of LA on macrophage polarization was not related to its acidification effect because the extracellular and intracellular pH values in macrophages were not different compared with the control (1 μM HCl). LDHA can be directly phosphorylated at Y10 at tyrosine residues by SRC kinase, which can be disturbed by the HIF-1α inhibitor PX478 48, 49. As expected, our results show that LA upregulated SRC kinase activity, resulting in LDHA phosphorylation at Y10 but not at Y239. Finally, it triggers the transcriptional expression of VEGF under normoxia and INOS under hypoxia to regulate macrophage polarization. Therefore, the SRC/LDHA pathway is a potential target for LA to regulate macrophage polarization during early pregnancy and might be used as a potential target for the rescue of M1 macrophage polarization in RPL.

AZD3965, MCT-1 inhibitor, is preferred to be used for the treatment of some tumors due to its selective inhibition of LA intake. In this study, we found that AZD3965 could decrease the embryo resorption rate and Mct-1 protein expression in AP mice. This may be due to the promotion and beneficial effects of LA at an appropriate level on embryo implantation and placentation. Hence, blockade of LA intake could rescue the pregnancy in AP mouse model and MCT-1 might be served as a potential therapeutic target for RPL.

Although we thoroughly investigated the mechanism of LA orchestrating decidual macrophage differentiation in early pregnancy, there are still some limitations in this study that could be addressed in future research. Firstly, the oxygen concentration in the decidua of women with NP and RPL was not measured directly. Secondly, it cannot be ruled out other cells in the decidua can also contribute to the high level of LA in the decidua of women with RPL, such as decidual stromal cells. Thirdly, co-culture of trophoblasts and macrophages should be conducted in addition to treating the macrophages with TCM.

In summary, we discovered the dual regulatory effects of trophoblast-derived LA on the polarization of pregnancy-related macrophages. The effects are related to the metabolic reprogramming in HIF-1α independent manner under normoxia or HIF-1α dependent manner under hypoxia via the SRC/LDHA pathway, which leads to the increased expression of VEGF and iNOS, respectively. The present study provides new insights into clarifying the internal connection among decidual macrophage polarization, oxygen environment and metabolic alterations in the decidua of early pregnancy in NP and RPL. Moreover, this study indicates that the maternal-fetal microenvironment in RPL is characterized by enhanced LA accumulation and exaggerated hypoxia. Blockade of LA intake with AZD3965 (MCT-1 inhibitor) may serve as a potential targeting strategy for correcting abnormal polarization of decidual macrophages and restoring immune tolerance in pregnancy-related diseases, including RPL. Nevertheless, more evidence in large sample sizes and deeper mechanisms are needed for further confirmation.

## Supplementary Material

Supplementary figure and tables.

## Figures and Tables

**Figure 10:**
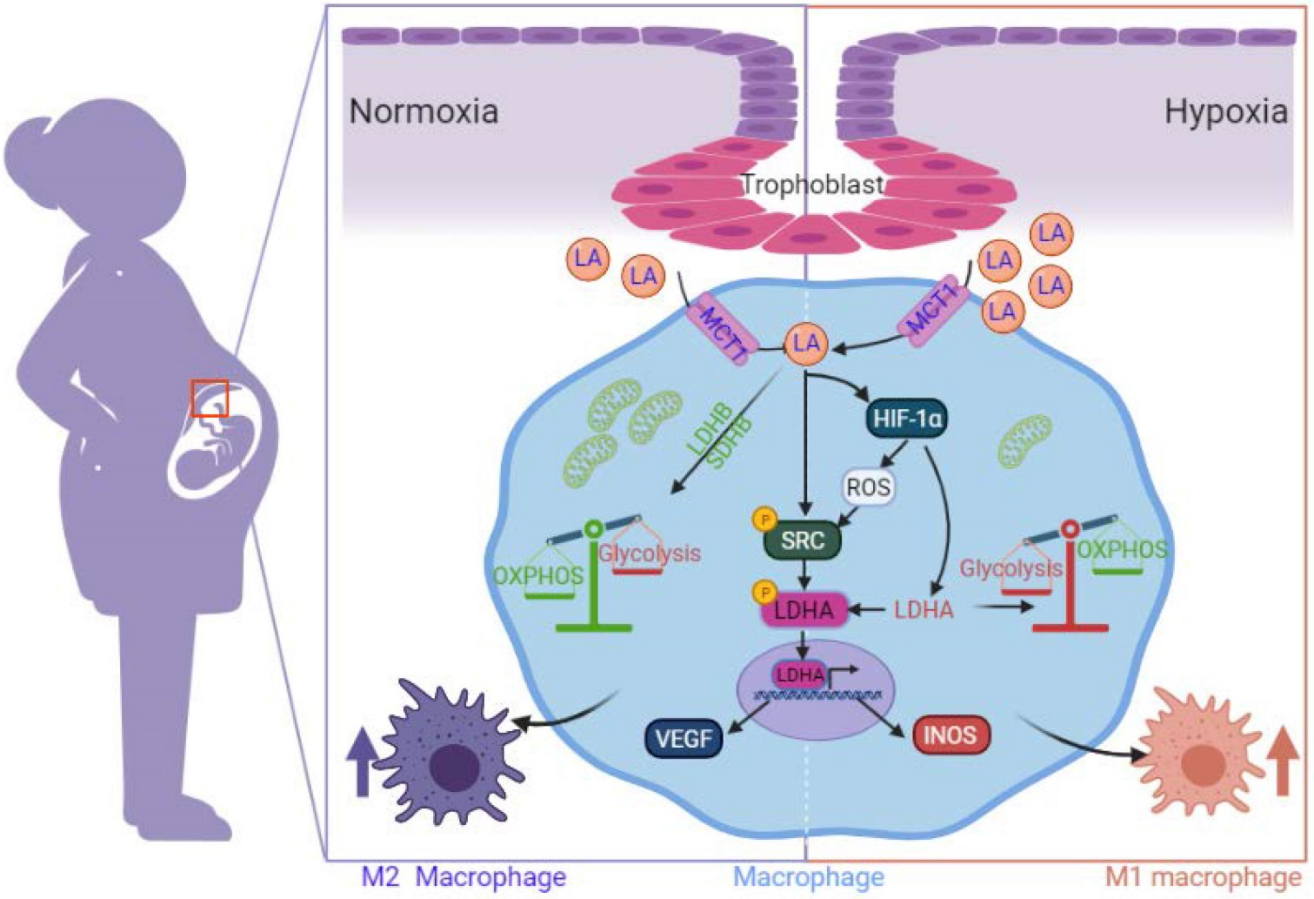
Schematic diagram of the mechanism by which trophoblast-derived LA regulates macrophage polarization under normoxia and hypoxia. Under normoxia, trophoblast-derived LA promotes macrophage polarization to the M2 phenotype by upregulating mitochondrial OXPHOS (especially increasing the expression of SDHB, COX4I1 and LDHB) and SRC-mediated LDHA phosphorylation (Y10) / VEGF expression. Under hypoxia, trophoblast-derived LA promotes macrophage polarization to the M1 phenotype by upregulating glycolysis mediated by HIF-1α, especially by increasing LDHA.

## Abbreviations

AP: abortion-prone
ARG1: arginase 1
ATP5F1A & B: ATP synthase F1 subunit
COX4I1 & 2: cytochrome c oxidase subunit 4 isoform
ERK: extracellular signal-regulated kinase
GLUT: glucose transporter
GPR: G protein-coupled receptors
HIF: hypoxia-inducible factor
ICM: inner cell mass
iNOS: inducible nitric oxide synthase
LA: lactic acid
LDH: lactate dehydrogenase
M1: classically activated macrophage
M2: alternatively activated macrophage
MCT: monocarboxylate transporter
NDUFA1 & 2: NADH dehydrogenase [ubiquinone] 1 alpha subcomplex subunits
NP: normal pregnancy
OXPHOS: oxidative phosphorylation
PBMC: peripheral blood mononuclear cells
PDK: phosphoinositide-dependent kinase
pTB: primary trophoblasts
ROS: reactive oxygen species
RPL: recurrent pregnancy loss
RT-qPCR: real-time quantitative polymerase chain reaction
STAT3: signal transducer and activator of transcription 3
TCM: trophoblast - conditioned media
UQCRC1 & 2: cytochrome b-c1 complex subunits
VEGF: vascular endothelial growth factor
