# Supplementary material for: Trophoblast-derived Lactic Acid Orchestrates Decidual Macrophage Differentiation via SRC/LDHA Signaling in Early Pregnancy
Source: Int J Biol Sci. 2022 Jan 1;18(2):599–616. doi: 10.7150/ijbs.67816 (PMC8741856; doi:10.7150/ijbs.67816)
Supplement: Supplementary file 1 — Supplementary figure and tables. [file ijbsv18p0599s1.pdf]

Supplementary Material

Trophoblast-derived lactic acid orchestrates decidual macrophage differentiation via SRC/LDHA signaling in early pregnancy

Lu Gao[1,#], Qian-Han Xu[1,#], Li-Na Ma[1,#], Jing Luo[1], Kahindo P. Muyayalo[1], Li-Ling Wang[1], Dong-Hui Huang[1], Xian-Jin Xiao[1], Shi-Bin Cheng[2], Gil Mor[1,3], Ai-Hua Liao[1,*]

[#] These authors have contributed equally to this work.

*** Correspondence:** Ai-Hua Liao, MD, PhD: E-mail: aihua_liao@sina.com

1. Supplementary Tables

Table S1 Primers used for quantitative real-time PCR

Gene	Primer sequences (5’ to 3’)	
ACTB	Forward,	GTTGCGTTACACCCTTTCTTGAC
	Reverse,	CTCGGCCACATTGTGAACTTTG
GLUT1	Forward,	ACCACCTCACTCCTGTTACTTACCT
	Reverse,	CTTACTTCTGTCTCACTCCCATCCA
GLUT4	Forward,	CTGGGCCTCACAGTGCTAC
	Reverse,	GTCAGGCGCTTCAGACTCTT
HK2	Forward,	GAGCCACCACTCACCCTACTG
	Reverse,	AGCCCATTGTCCGTTACTTTCAC
GPI	Forward,	GGAAGGGGTACACAGGCAAG
	Reverse,	TGGAGAAACCACTCCTTCGC
PFKL	Forward,	GCTGCAAGGCCTTTACCACC
	Reverse,	CCAGCCTCTCACACATGAAGT
ALDOA	Forward,	AGATCCTCCCTGATGGGGAC
	Reverse,	CTTCTGAGTGCAAGCATGGC
TPI	Forward,	ATGGCTGAAGTCCAACGTC
	Reverse,	AAGGAAGCCATCCACATCAG
GAPDH	Forward,	TGCACCACCAACTGCTTAGC
	Reverse,	GGCATGGACTGTGGTCATGAG
PGK1	Forward,	CCCACAGCTCCATGGTAGGA
	Reverse,	TTGGCCAGTCTTGGCATTCT
PGAM1	Forward,	TCGCTCTCTTCTGCACTGAG
	Reverse,	ACCTGGAGAACCGCTTCAG
ENO1	Forward,	GCCTCCTGCTCAAAGTCAAC
	Reverse,	AACGATGAGACACCATGACG
ENO2	Forward,	GAAGCCATCCAAGCGTGCAA
	Reverse,	AAAGTGCGGAACCCCAATGA
PKM2	Forward,	AGAACTTGTGCGAGCCTCAA
	Reverse,	GGCCTTGCCAACATTCATGG
LDHA	Forward,	ACGTGCATTCCCGATTCCTT
	Reverse,	GGAAAAGGCTGCCATGTTGG
LDHB	Forward,	TCCGCACGACTGTTACAGAG
	Reverse,	TCAGCCAGAGACTTTCCCAG
MCT1	Forward,	AGGTCCAGTTGGATACACCCC
	Reverse,	GCATAAGAGAAGCCGATGGAAAT
MCT4	Forward,	TCTGAAGGGGGACAGGTGAG
	Reverse,	ATGGAGGAGATCCAGGCTGT

NDUFA1	Forward,	CACTAACGGGGGCAAGGAAA
	Reverse,	CTTCTAGCAGGGGTAGATGGC
NDUFA2	Forward,	CGCATCCACTTATGTCAGCG
	Reverse,	TGGCCAAATGCGTAGCGG
SDHA	Forward,	CGAACGTCTTCAGGTGCTTT
	Reverse,	AAGAACATCGGAACTGCGAC
SDHB	Forward,	CACAGATGCCTTCTCTGCAT
	Reverse,	CGAACGTCTTCAGGTGCTTT
UQCRC10	Forward,	TTGTACTCCCTGCTGTTCCG
	Reverse,	TGTTTCCACAGCTTCCCCTC
UQCRC11	Forward,	CTACCGGGAGCTGGTCAAGA
	Reverse,	GTACCCAGTCCAGGATCAGC
UQCRC1	Forward,	GCATCTGGCTTTCAAGGGAA
	Reverse,	GTGTGCTGAGGTACTCGGTC
UQCRC2	Forward,	TTTGCATGCAGCAGCTTACC
	Reverse,	GGATGACTCACACCAAGTCCA
COX4I1	Forward,	TGTGTACGAGCTCATGAAAGTGT
	Reverse,	CGATGAAGAACATGGCACCG
COX4I2	Forward,	ATGAACCGTCGCTCCAATGA
	Reverse,	AAATACGTAGACCCGCTGCC
ATP5F1A	Forward,	GGAGCTGTTGGGTCGTGTAG
	Reverse,	GGTTTTCCCAGTCTGTCGGT
ATP5F1B	Forward,	GAAGGCTTGGTTAGAGGCCA
	Reverse,	AGCACCACCAAAAAGCCCAAT
IL6	Forward,	CCTCCAGAACAGATTTGAGAGTAGT
	Reverse,	GGGTCAGGGGTGGTTATTGC
IL10	Forward,	CAACCTGCCTAACATGCTTCGAG
	Reverse,	TCTCAGCTTGGGGCATCACCT
TNFA	Forward,	AGGACACCATGAGCACTGAAAGC
	Reverse,	AAGGAGAAGAGGCTGAGGAACAAG
IL1B	Forward,	GAAATGATGGCTTATTACAGTGGCA
	Reverse,	GTAGTGGTGGTCGGAGATTCGTAG
TGFB	Forward,	GAAACCCACAACGAAATCTATGAC
	Reverse,	ACGTGCTGCTCCACTTTTAACT
ARG1	Forward,	TAACTCGAACAGTGAACACAGCAG
	Reverse,	TAGGTGGGTTAAGGTAGTCAATAGG

2. Supplementary Figures

Figure S1.

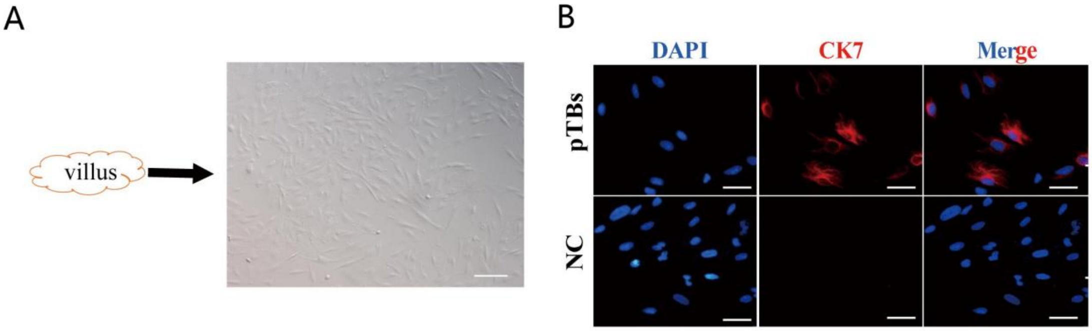

Figure S1. Isolation and identification of primary first-trimester trophoblasts. (A) Primary first-trimester trophoblasts were isolated from villi, identified under an optical microscope. (B) Primary first-trimester trophoblasts were stained for CK7 by immunofluorescence pTBs: primary first-trimester trophoblasts. NC: negative control; DAPI: 4',6-diamidino-2-phenylindole; CK7: cytokeratin7; Scale bars, 10 μm for optical microscopy; 25 μm for immunofluorescence.

Figure S2.

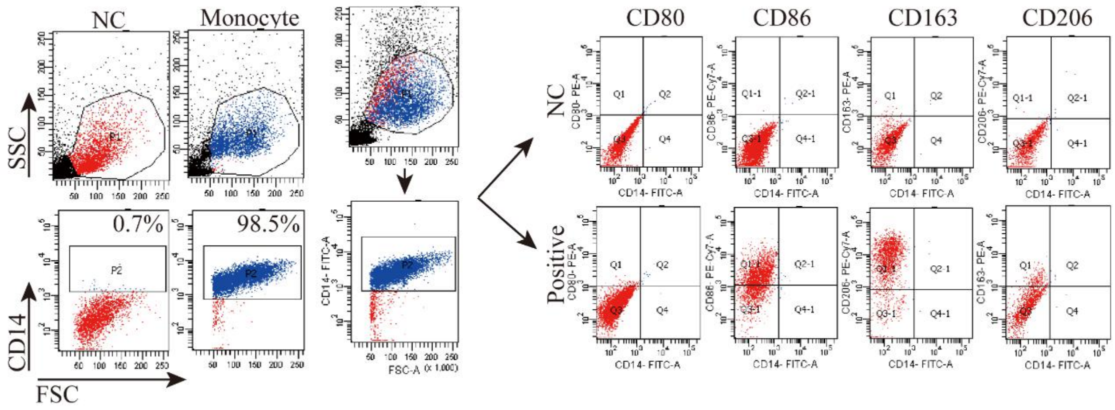

Figure S2. Flow cytometric gating strategy to identify macrophages and trophoblasts. Macrophages isolated from the PBMCs of first-trimester pregnant women were cultured for 7 days with supplemental rhM-CSF. Dot plot of forward scatter (FSC) versus side scatter (SSC) for all events, with the population of $CD14^+$ macrophages enclosed. In $CD14^+$ gates, CD80, CD86, CD163 and CD206 were analyzed.

Figure S3.

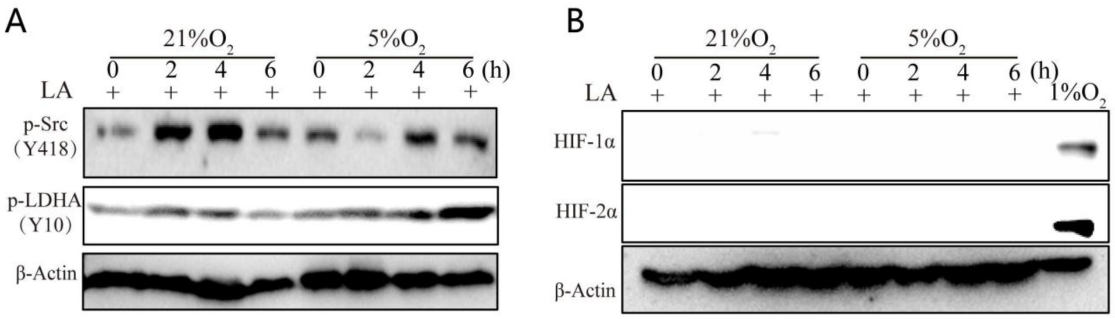

Figure S3. Protein expression of p-Src (Y418), p-LDHA (Y10), HIF-1α and HIF-2α by western blotting assay in cells treated with LA for 0-6 h under 21% O_2 and 5% O_2. 1% O_2 was used as a positive control.

Figure S4.

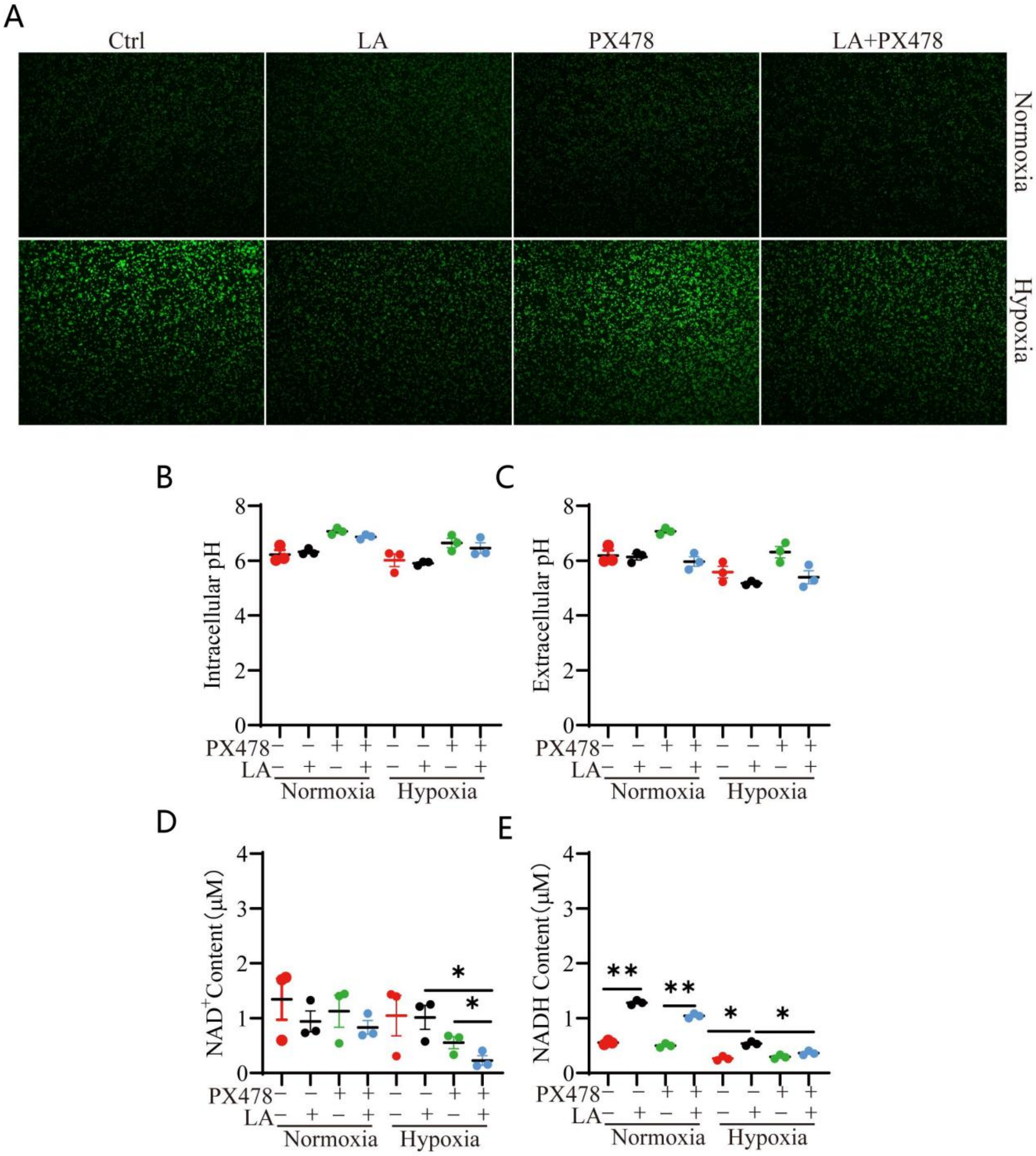

Figure S4. ROS fluorescence intensity, pH value, NAD^+ and NADH content in macrophages treated with or without LA in the presence of PX478 under normoxia and hypoxia. (A) Representative staining of ROS by immunofluorescence in macrophages

treated with or without LA in the presence of PX478 under normoxia and hypoxia (n = 3). (B) Intercellular pH in macrophages treated with or without LA in the presence of PX478 under normoxia and hypoxia (n = 3). (C) Extracellular pH in macrophages treated with or without LA in the presence of PX478 under normoxia and hypoxia (n = 3). (D) NAD^+ content in macrophages treated with or without LA in the presence of PX478 under normoxia and hypoxia (n = 3). (E) NADH content in macrophages treated with or without LA in the presence of PX478 under normoxia and hypoxia (n = 3). Data are shown as the mean ± SEM. *$p < 0.05$, **$p < 0.01$.

Figure S5.

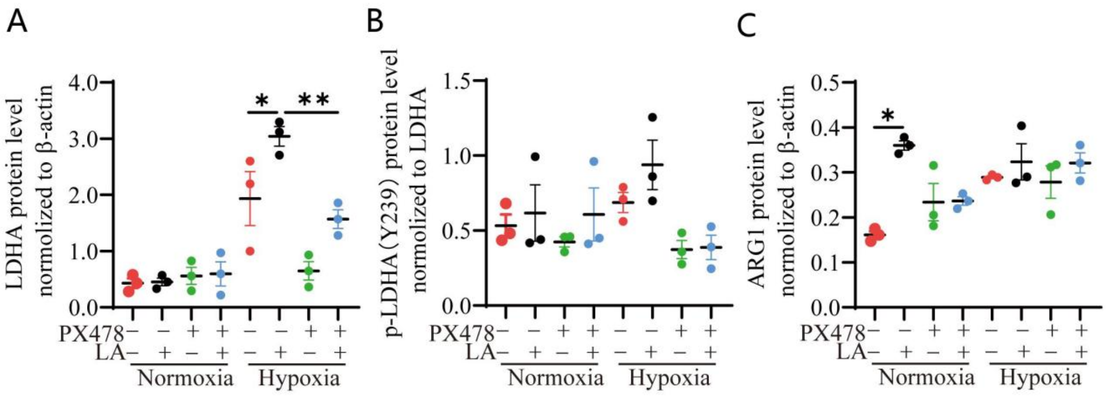

Figure S5. Quantification of p-LDHA (Y239), LDHA and ARG1 protein expression in cells treated with or without LA in the presence of PX478 under normoxia and hypoxia (n = 3). Data are shown as the mean ± SEM. *$p < 0.05$, **$p < 0.01$.

Figure S6.

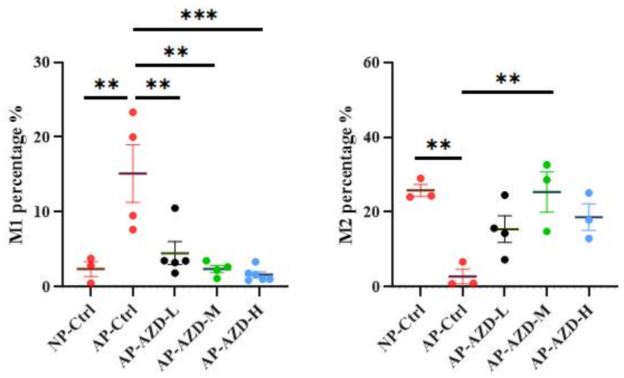

Figure S6. The statistical data of the percentages of M1 ($CD14^+$ $CD86^+$ $CD206^-$) and M2 ($CD14^+$ $CD86^-$ $CD206^+$) macrophages in the decidua of all mice groups. Data are shown as the mean ± SEM. $^{**}p < 0.01$, $^{***}p < 0.001$.
